# Immunoglobulin G *N*-glycosylation predicts outcome in sepsis caused by pathogenic Gram-negative bacteria and Gram-positive bacteria: a nested case-control study

**DOI:** 10.3389/fimmu.2026.1826457

**Published:** 2026-06-23

**Authors:** Huachen Wang, Yan Zhang, Anlu Ouyang, Tao Wang, Hongda Hou, Haifeng Hou, Bing Chen

**Affiliations:** 1 Institute of Infectious Diseases, The Second Hospital of Tianjin Medical University, Tianjin, China; 2 Intensive Care Unit, The Second Hospital of Tianjin Medical University, Tianjin, China; 3 School of Public Health, Shandong First Medical University & Shandong Academy of Medical Sciences, Jinan, China

**Keywords:** biomarker, glycosylation, immunoglobulin G, inflammation, sepsis

## Abstract

**Background:**

Sepsis remains a critical global health challenge with high mortality. Rapid distinction between Gram-positive and Gram-negative pathogens is critical for empiric antibiotic selection, yet reliable biomarkers for such pathogen stratification are lacking. Immunoglobulin G (IgG) *N*-glycosylation modulates inflammatory responses in various diseases, suggesting its potential role in sepsis pathogenesis and prognosis.

**Methods:**

In this nested case-control study, 180 septic patients (100 Gram-negative, 80 Gram-positive) and 100 healthy controls were enrolled. IgG *N*-glycosylation was analyzed using Hydrophilic interaction chromatography based on ultra-performance liquid chromatography (HILIC-UPLC). Inflammatory cytokines and clinical parameters were collected. Least absolute shrinkage and selection operator (LASSO) and logistic regression were used to identify glycan biomarkers and construct predictive models for pathogen type and 90-day mortality. Model performance was evaluated using the area under the receiver operating characteristic curve (AUC).

**Results:**

In patients with Gram-negative sepsis, levels of 12 glycan peaks (A2B, M5, A2G1, FA2[3]G1, FA2[3]BG1, A2G2, A2BG2, FA2G2, A2G2S1, FA2G2S1, A2G2S2, A2BG2S2) were significantly decreased, while levels of FA1, A2, FA2, FA2B, FA2BG2, and FA2FG2S1 were increased (all *P* < 0.05, *q* < 0.05), compared to patients with Gram-positive sepsis. A model incorporating glycan peaks with routine clinical markers showed excellent discrimination (AUC = 0.931 in training, 0.917 in validation). Significant differences in fucosylation, sialylation, agalactosylation (G0), and digalactosylation (G2) levels were observed between septic survivors and septic non-survivors. For 90-day mortality, glycan FA2 was a strong independent predictor (AUC = 0.792), outperforming the SOFA score (AUC = 0.673). A combined model of FA2 and SOFA score improved predictive accuracy (AUC = 0.820).

**Conclusion:**

IgG *N*-glycosylation profiles serve as effective biomarkers for distinguishing pathogenic Gram-negative from Gram-positive sepsis and for predicting mortality. Integration of glycan peaks with clinical scores enhances risk stratification, highlighting their utility as a complementary tool in sepsis prognostication.

## Background

Sepsis is a systemic reaction with dysregulated response to infection (1). It is associated with severe organ dysfunction, including lung, liver, and kidney dysfunction, leading to cognitive impairment and septic shock (2). The common pathogens associated with sepsis are Staphylococcus and Escherichia coli, which are typically acquired during hospitalization (3, 4). Sepsis can be triggered by both Gram-negative and Gram-positive bacteria, which collectively represent a leading cause of this condition. Although the incidence of sepsis decreased by 37.0% from 1990 to 2017, the estimated value of all-cause sepsis-related deaths is 21.4 million, representing 31.5% of total global deaths in 2021 (5, 6). Sepsis imposed a heavy burden on society, posing a major challenge to public health systems (7).

Glycosylation is a crucial post-translational modification that occurs on more than 50% of proteins within cells (8). Immunoglobulin G (IgG) is a central component of humoral immunity and one of the most abundant *N*-glycoproteins in human plasma (9, 10). The IgG fragment crystallizable (Fc) region contains conserved *N*-linked glycosylation sites at asparagine 297 (Asn-297), where specific glycan–protein and glycan–glycan interactions play a critical role in modulating antibody conformation (11). IgG glycosylation modulates IgG effector functions, thereby affecting pro- and anti-inflammatory signaling and cellular immune response (12). Specifically, alterations in sialylation and galactosylation levels on IgG *N*-glycans profoundly modulate IgG structure and effector functions (12, 13). Previous studies showed that abnormal *N*-glycosylation strongly interferes with IgG function and is associated with inflammation-associated diseases or cancers (9, 14–17).

While the clinical manifestations and severity of sepsis vary according to the causative pathogen, reliable indicators to differentiate Gram-negative from Gram-positive infections are still lacking (18). IgG *N*-glycans are related to inflammatory response and risk factors of various diseases (19). Evidence suggests an association between IgG *N*-glycans and sepsis (20). We investigated differences in IgG *N*-glycans and inflammatory factors between Gram-negative and Gram-positive sepsis. Moreover, a model of glycan peaks was developed to predict mortality in patients. This study may provide new biomarkers for distinguishing between Gram-negative and Gram-positive bacterial sepsis.

## Methods

### Study samples

**Figure 1:**
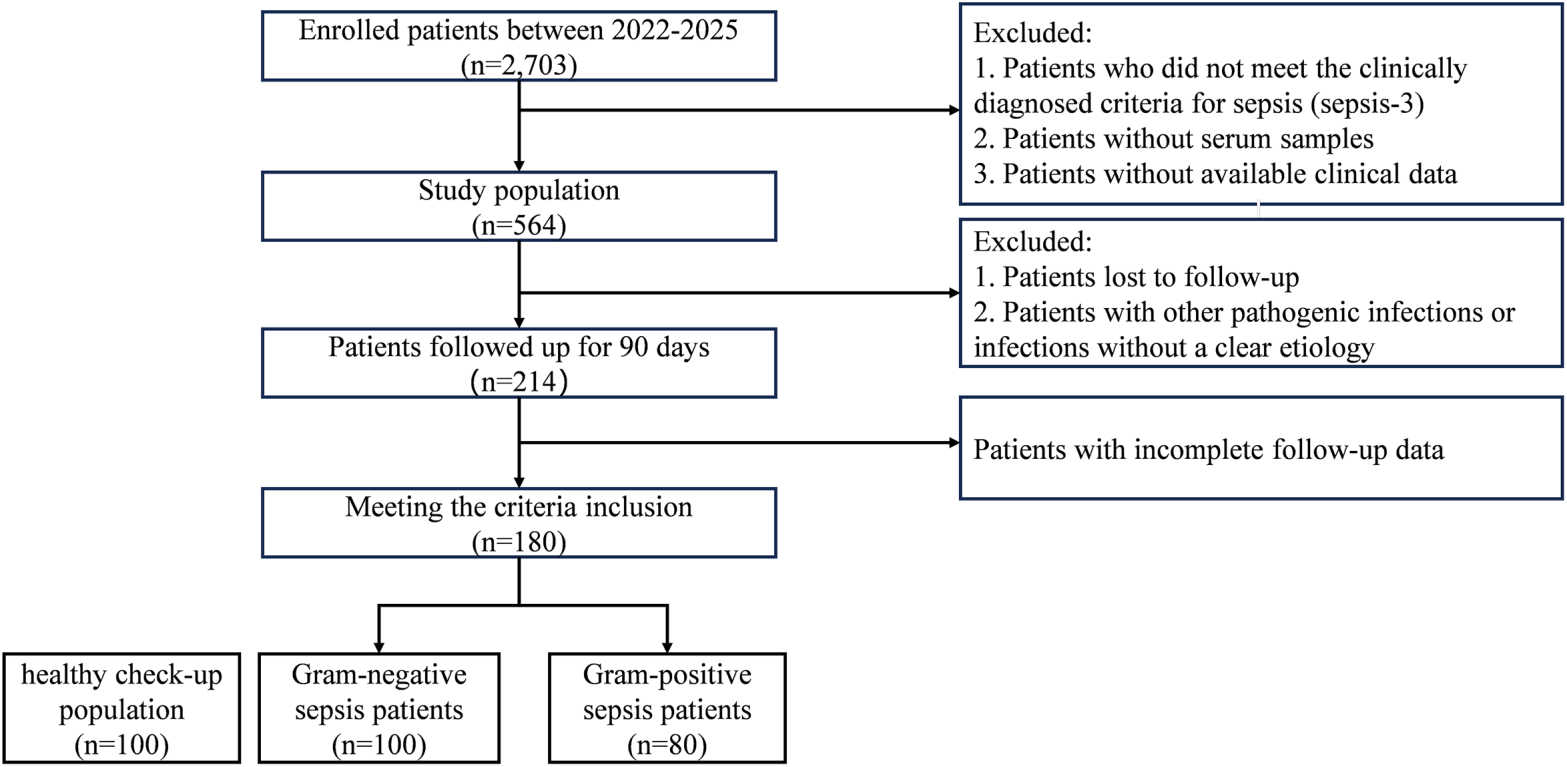
Flow diagram of participants in the study.

The study was a nested case-control study design. From 2022-2025, a total of 2,703 septic patients were enrolled in the Second Hospital of Tianjin Medical University. Out of these patients, 564 patients met the following criteria:(1) available clinical data; (2) were diagnosed with sepsis according to the Third International Consensus Definitions (Sepsis-3) (21); (3) were hospitalized with available serum samples. The exclusion criteria were: (1) lack of available clinical data; (2) pregnancy or lactation; (3) non-infectious causes of organ dysfunction; (4) an unclear baseline or a Sequential Organ Failure Assessment (SOFA) score of less than 2. Finally, 180 septic patients, comprising 100 with Gram-negative and 80 with Gram-positive infections, were enrolled. The details of inclusion were shown in **Figure 1**. 100 healthy controls were also selected for analysis. All participants signed informed consent forms. This study was approved by the Clinical Research Ethics Committee of the Second Hospital of Tianjin Medical University and conducted in accordance with the Declaration of Helsinki (22). Written informed consent has been obtained from each patient or their immediate family members.

### Blood sample collection and preservation

5 mL fasting blood samples were taken into Vacutainer blood collection tubes and separated by centrifugation. After processing, the supernatant liquid was aliquoted into 1.5 mL cryovials. All samples were stored in -80 °C before laboratory analysis.

### Baseline data collection

Data on demographic characteristics and relevant epidemiological factors were gathered through participant interviews. Demographic traits include age and gender. The following baseline parameters were obtained through retrospective review of clinical records and examinations: (a) hematological indices: white blood cell (WBC), absolute neutrophil count (NEUT), platelet (PLT), and mean platelet volume (MPV); (b) hepatic enzymes: alanine aminotransferase (ALT) and aspartate aminotransferase (AST); and (c) the SOFA score.

### IgG N-glycans analysis

We detected IgG *N-*glycans in serum from sepsis patients using methods reported by previous studies (23–25). IgG purification was performed first using a protein G monolithic plate (BIA Separations, Slovenia). The IgG sample was eluted with formic acid and subsequently neutralized with ammonium bicarbonate. *N*-glycans were then liberated from the IgG sample by incubation with PNGase F. We used a solution reagent with 2-aminobenzamide to label *N*-glycans. The hydrophilic interaction chromatography based on ultraperformance liquid chromatography (HILIC-UPLC) analysis of the labeled glycan peaks was carried out by the Waters ACQUITY UPLC system (25). **Supplementary Figures 1**-**3** show chromatograms presenting the differences in *N*-glycan peaks between patients with sepsis and control individuals. Chromatograms were integrated to 24 peaks based on standardized baseline and valley settings. The level of each glycan was quantified as the percentage of its peak area relative to the total integrated area of all IgG glycan peaks (26–28). From the initial glycan peak areas, we derived 54 quantitative glycan traits, which encompass four major IgG *N*-glycosylation features: fucosylation, sialylation, bisecting GlcNAc, and galactosylation (**Supplementary Table 1**).

### Analysis of inflammatory cytokines

We detected the levels of inflammatory cytokines in serum using flow cytometry with a Weimi Bio-Tech Panel kit. The following key inflammatory cytokines were included in the analysis: interleukin-2 (IL-2), interleukin-4 (IL-4), interleukin-6 (IL-6), interleukin-10 (IL-10), tumor necrosis factor-α (TNF-α), and interferon-γ (IFN-γ).

### Statistical analysis

In our study, categorical variables were summarized as frequency (percent) and compared using Pearson’s chi-squared test (χ² test). The Kolmogorov-Smirnov test was applied to test the continuous variables for normality. Continuous variables were reported as mean ± standard deviation (SD) if normally distributed, or as median and interquartile range (IQR) otherwise. Normally distributed continuous variables were compared using one-way analysis of variance (ANOVA). For non-normally distributed variables, we employed the Mann-Whitney U test to assess the differences between groups. To explore the association between sepsis pathogens and glycan peaks, we first performed univariate regression to identify significant glycan peaks, which were then entered into a multivariate logistic regression model to assess independent associations.

Based on methodologies established in previous studies, the patients were randomly split into training and internal validation sets at a 7:3 ratio for internal performance assessment (29, 30). The training set included 126 participants, and the validation set 54. We employed the least absolute shrinkage and selection operator (LASSO) regression to select original glycan peaks. Eligible glycan peaks were identified as biomarkers for differentiating Gram-negative from Gram-positive bacterial infections. Predictive models for sepsis features and 90-day mortality were then constructed using the selected glycan peak biomarkers. The performance of these models was evaluated with the receiver operating characteristic (ROC) curve, the area under the curve (AUC), and 95% confidence intervals. A predictive nomogram incorporating the specific glycan peaks and baseline parameters was created to visualize the probability of different bacterial infections, and its calibration was assessed by the Hosmer-Lemeshow test.

All statistical analyses were performed using SPSS 26.0 (IBM, Armonk, NY, USA) and R 4.2.1 (R Foundation for Statistical Computing), with statistical significance set at *P* < 0.05.

## Results

### Characteristics of patients with sepsis

In this nested case-control study, a total of 100 patients with Gram-negative sepsis and 80 patients with Gram-positive sepsis were included. The levels of WBC, NEUT, AST, and ALT were higher in patients with Gram-negative sepsis than in those with Gram-positive sepsis. There were no significant differences between the two groups in terms of Age, Gender, PLT, and MPV (**Table 1**).

**Table 1:** Characteristics of the patients with sepsis.

Variables	Patients with Gram-negative sepsis (n = 100)	Patients with Gram-positive sepsis (n = 80)	*P*	*q*
Age(years)	69.50 (61.00, 77.00)	68.00 (60.00, 77.25)	0.783	0.831
Gender [n(%)]			0.676	0.831
men	52(52)	45(56.25)		
women	48(48)	35(43.75)		
WBC (10*9/L)	18.18 (11.80, 22.58)	11.43 (7.20, 17.60)	4.326E-05	4.326E-04
NEUT (10*9/L)	91.25 (87.27, 93.53)	88.15 (82.15, 93.35)	0.011	0.027
PLT (10*9/L)	186.00 (111.50, 270.75)	193.00 (106.25, 340.25)	0.340	0.567
MPV (fL)	10.00 (9.30, 11.53)	10.15 (9.38, 11.40)	0.831	0.831
AST(U/L)	40.15 (25.00, 109.60)	27.70 (17.67, 48.45)	8.393E-04	2.798E-03
ALT(U/L)	30.65 (19.67, 61.65)	20.90 (13.30, 34.90)	5.003E-04	2.502E-03
SOFA	8.00 (6.00, 10.00)	8.00 (6.00, 10.25)	0.677	0.831
90-day outcome[n(%)]			0.067	0.135
Non-survivors	39(39)	20(25)		
Survivors	61(61)	60(75)		

WBC, white blood cell; NEUT, absolute neutrophil count; PLT, platelet; MPV, Mean Platelet Volume: ALT, alanine aminotransferase; AST, aspartate aminotransferase; SOFA, Sequential Organ Failure Assessment; FDR, false discovery rate; Data are presented as n (%) for categorical variables and median (IQR) for continuous variables. *P* < 0.05 was considered statistically significant; *q* < 0.05 was considered statistically significant after correction using FDR.

### Inflammatory cytokines

The serum samples collected from the patients with sepsis were analyzed to assess the levels of six cytokines. As shown in **Supplementary Table 2**, no significant differences were observed for IL-2, IL-4, IL-6, IL-10, TNF-α, and IFN-γ between the groups (all *q* > 0.05).

### The IgG glycome composition of the patients with sepsis

A total of 24 initial glycan peaks were detected using the HILIC-UPLC assay (**Table 2**, **Supplementary Figure 4**). The abundance of 18 glycan peaks showed significant differences between the groups. Specifically, compared with patients with Gram-positive sepsis, the levels of 12 glycan peaks (i.e., A2B, M5, A2G1, FA2[3]G1, FA2[3]BG1, A2G2, A2BG2, FA2G2, A2G2S1, FA2G2S1, A2G2S2, and A2BG2S2) were significantly decreased in patients with Gram-negative sepsis (*P* < 0.05, *q* < 0.05). By contrast, the levels of FA1, A2, FA2, FA2B, FA2BG2, and FA2FG2S1 were increased in patients with Gram-negative sepsis (*P* < 0.05, *q* < 0.05). In addition, we calculated 54 derived glycan traits based on the initial glycan measurements; 31 of the 54 derived glycan traits exhibited differences between the groups. The detailed results of the derived traits are provided in **Supplementary Table 3**.

**Table 2:** Comparisons of 24 initial glycan peaks in patients with sepsis.

Glycan peaks	Patients with Gram-negative sepsis (n = 100)	Patients with Gram-positive sepsis (n = 80)	*P*	*q*
FA1	0.36 (0.25, 0.53)	0.25 (0.18, 0.31)	1.510E-06	7.249E-06
A2	0.83 (0.52, 1.29)	0.61 (0.36, 0.90)	1.105E-03	2.029E-03
A2B	0.19 (0.14, 0.31)	0.28 (0.22, 0.36)	5.649E-05	1.695E-04
FA2	27.42 (22.98, 32.59)	24.81 (22.87, 28.39)	0.021	0.030
M5	0.10 (0.08, 0.14)	0.16 (0.11, 0.20)	4.408E-07	3.526E-06
FA2B	5.66 (4.61, 6.52)	4.91 (4.03, 5.44)	6.361E-06	2.544E-05
A2G1	0.45 (0.32, 0.57)	0.57 (0.43, 0.77)	1.051E-04	2.541E-04
FA2[6]G1	17.40 (16.28, 18.17)	16.95 (16.17, 18.06)	0.328	0.369
FA2[3]G1	9.25 (7.99, 10.02)	10.21 (9.59, 11.08)	2.466E-07	3.526E-06
FA2[6]BG1	4.29 (3.77, 4.79)	4.48 (3.89, 5.02)	0.228	0.274
FA2[3]BG1	0.78 (0.69, 0.90)	0.90 (0.80, 1.04)	1.127E-05	3.862E-05
A2G2	0.54 (0.38, 0.81)	0.73 (0.56, 0.99)	1.059E-04	2.541E-04
A2BG2	0.28 (0.24, 0.35)	0.34 (0.25, 0.42)	0.014	0.022
FA2G2	9.96 (7.75, 11.93)	12.18 (10.90, 13.24)	3.752E-07	3.526E-06
FA2BG2	1.42 (1.32, 1.62)	1.31 (1.09, 1.54)	4.098E-03	7.025E-03
FA2G1S1	3.27 (2.68, 3.60)	3.16 (2.76, 3.55)	0.432	0.451
A2G2S1	0.96 (0.85, 1.07)	1.10 (0.89, 1.32)	2.362E-03	4.361E-03
FA2G2S1	7.57 (6.17, 9.17)	9.33 (8.08, 10.34)	1.239E-06	7.249E-06
FA2BG2S1	2.21 (1.98, 2.48)	2.18 (1.87, 2.54)	0.840	0.840
FA2FG2S1	0.16 (0.13, 0.21)	0.12 (0.07, 0.20)	2.296E-04	5.010E-04
A2G2S2	0.83 (0.73, 0.96)	0.94 (0.70, 2.29)	0.020	0.030
A2BG2S2	0.22 (0.18, 0.28)	0.24 (0.19, 0.42)	0.027	0.036
FA2G2S2	1.92 (1.69, 2.40)	1.89 (1.59, 2.21)	0.338	0.369
FA2BG2S2	2.52 (2.14, 3.07)	2.48 (2.16, 2.71)	0.116	0.146

Initial glycans compositions: F at the start of the abbreviation indicates a core-fucose α 1,6-linked to the inner GlcNAc; Mx, number (x) of mannose on core GlcNAcs; Ax, number of antenna (GlcNAc) on trimannosyl core; A2, biantennary with both GlcNAcs as *β* 1,2-linked; B, bisecting GlcNAc linked β1,4 to β 1,3 mannose; G (x), number (x) of *β* 1,4 linked galactose on antenna; F (x), number (x) of fucose linked α 1,3 to antenna GlcNAc; S (x), number (x) of sialic acids linked to galactoses. FDR, false discovery rate; GP, glycan peak. *P* < 0.05 was considered statistically significant; *q* < 0.05 was considered statistically significant after correction using FDR.

Moreover, to explore the differential abundance patterns of IgG *N*-glycans between patients with Gram-negative and Gram-positive sepsis, we also calculated the abundances of the four major glycosylation features: fucosylation, bisecting GlcNAc, sialylation, and galactosylation (31). As shown in **Table 3** and **Supplementary Figure 5**, the patients with Gram-negative sepsis exhibited lower levels of both fucosylation (95.09% vs. 95.95%) and sialylation (19.57% vs. 22.22%) compared to the patients with Gram-positive sepsis. Regarding galactosylation, agalactosylation (G0) was detected at a level of 35.04% in patients with Gram-negative sepsis, which was statistically significantly higher than the 30.91% observed in patients with Gram-positive sepsis. The abundances of monogalactosylation (G1) and digalactosylation (G2) in patients with Gram-negative sepsis were 32.41% and 12.44%, respectively, which were lower than those found in patients with Gram-positive sepsis (33.41% and 14.55%). However, there was no statistical difference in the abundance of bisecting GlcNAc.

**Table 3:** Comparisons of the relative abundance of four IgG *N-*glycosylation features (%) in patients with sepsis.

Summary glycan peaks	Patients with Gram-negative sepsis (n = 100)	Patients with Gram-positive sepsis (n = 80)	*P*	*q*
Fucosylation	95.09 (94.20, 96.02)	95.95 (94.26, 97.71)	1.970E-03	2.364E-03
Bisecting GlcNAc	17.38 (15.90, 19.45)	16.87 (16.18, 17.72)	0.110	0.110
Sialylation	19.57 (17.89, 22.32)	22.22 (19.63, 24.69)	8.822E-05	2.647E-04
Galactosylation				
G0	35.04 (29.24, 40.62)	30.91 (28.44, 34.72)	9.901E-04	1.485E-03
G1	32.40 (30.68, 33.64)	33.31 (32.28, 34.66)	7.686E-04	1.485E-03
G2	12.44 (9.88, 14.48)	14.55 (12.90, 15.77)	1.867E-06	1.120E-05

FDR, false discovery rate; GlcNAc, N-acetylglucosamine; G0, agalactosylation; G1, monogalactosylation; G2, digalactosylation.*P* < 0.05 was considered statistically significant; *q* < 0.05 was considered statistically significant after correction using FDR.

### Association of IgG N-glycans with Gram-positive sepsis

**Figure 2:**
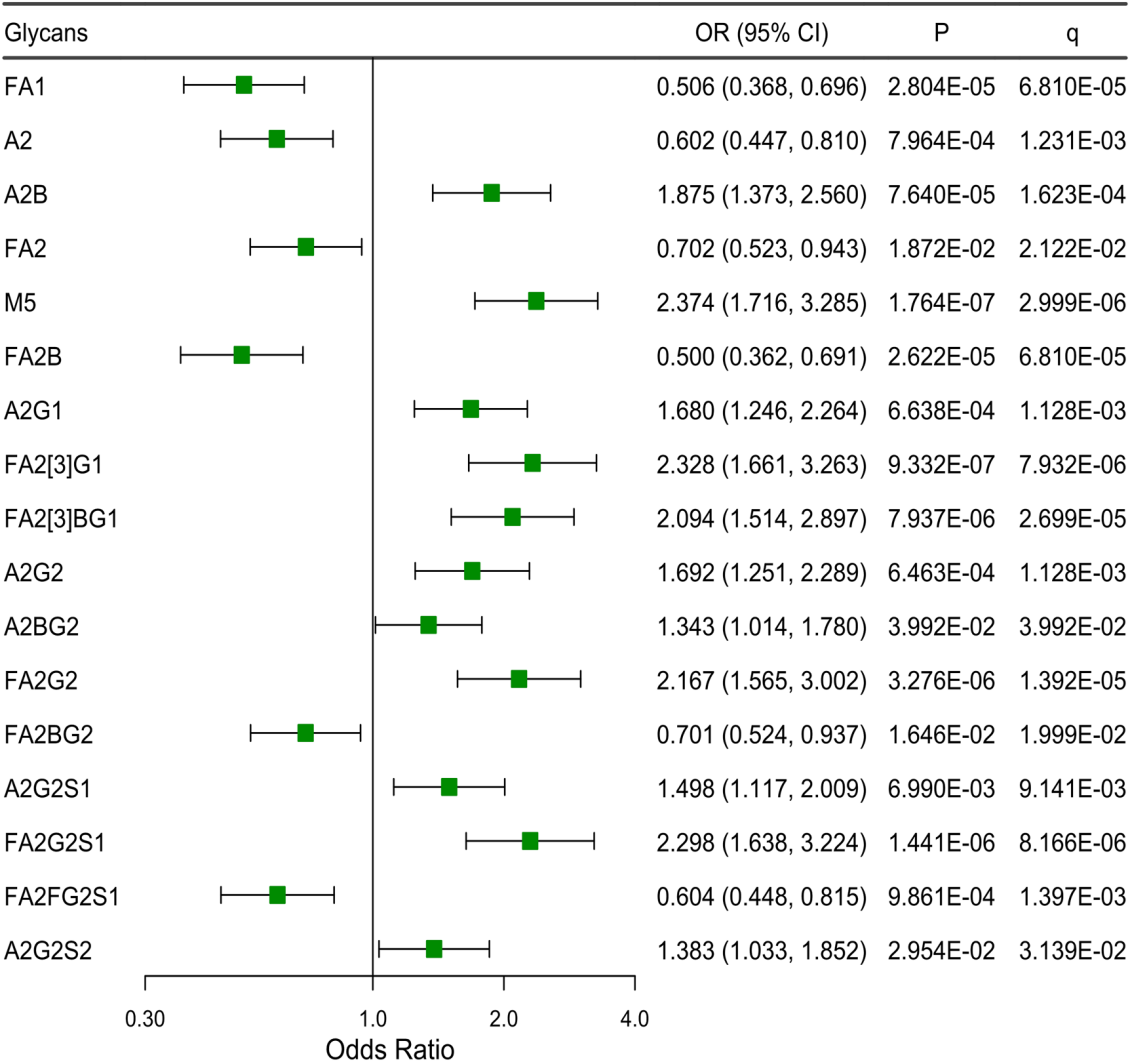
Forest plot of multivariable-adjusted associations between 24 initial glycan peaks and sepsis caused by Gram-negative bacteria and sepsis caused by Gram-positive bacteria. CI, confidence interval; FDR, false discovery rate; GP, glycan peak; OR, odds ratio. Initial glycans compositions: F at the start of the abbreviation indicates a core-fucose α 1,6-linked to the inner GlcNAc; Mx, number (x) of mannose on core GlcNAcs; Ax, number of antenna (GlcNAc) on trimannosyl core; A2, biantennary with both GlcNAcs as *β* 1,2-linked; B, bisecting GlcNAc linked β1,4 to β 1,3 mannose; G (x), number (x) of *β* 1,4 linked galactose on antenna; F (x), number (x) of fucose linked α 1,3 to antenna GlcNAc; S (x), number (x) of sialic acids linked to galactoses. Adjusted for age, gender, WBC, NEUT, AST, ALT. P < 0.05 was considered statistically significant, and q < 0.05 was considered statistically significant after correction using FDR.

The univariate analysis results of the 24 initial glycan peaks are presented in **Supplementary Table 4**. After adjusting for age, gender, WBC, NEUT, AST, and ALT, there were 17 initial glycan peaks significantly associated with Gram-positive sepsis (**Figure 2**, **Supplementary Table 5**). Additionally, **Supplementary Table 6** showed the univariate and multivariate logistic regression analysis results of the major glycosylation features. All features were statistically significant after adjusted for age, gender, WBC, NEUT, AST, and ALT: Fucosylation (OR = 1.297, 95% CI: 1.102, 1.551), Bisecting GlcNAc (OR = 0.838, 95% CI: 0.716, 0.971), Sialylation (OR = 1.184, 95% CI: 1.084, 1.303), G0 (OR = 0.919, 95% CI: 0.872, 0.964), G1 (OR = 1.288, 95% CI: 1.116, 1.518), G2 (OR = 1.335, 95% CI: 1.183, 1.528).

### Association between IgG N-glycans and inflammatory cytokines

**Supplementary Table 7** presented the Spearman correlation coefficients between the initial glycan peaks and circulating inflammatory cytokines. IL-6 exhibited a significant negative correlation with FA2FG2S1, A2G2S2, and A2BG2S2 (*P* < 0.05), whereas TNF-α demonstrated a positive correlation with M5 (*P* < 0.05).

### Discrimination of sepsis types based on IgG N-glycans

**Figure 3:**
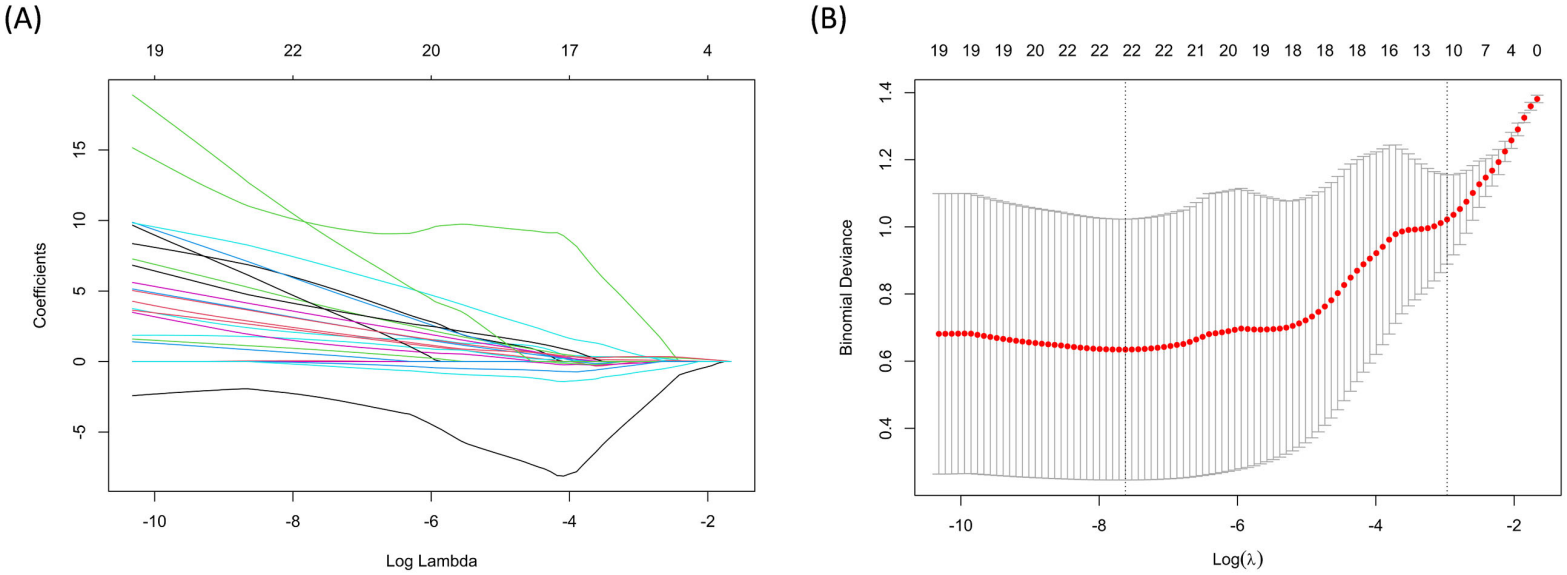
LASSO regression analysis for variable selection. **(A)** LASSO coefficient of 24 variables; **(B)** optimal penalty coefficient (λ = 0.05133141) in LASSO regression. LASSO, least absolute shrinkage and selection operator.

As shown in **Figure 3**, to establish a predictive model of sepsis, LASSO regression was first carried out to screen initial glycan peaks, which identified ten significant glycan peaks (FA1, A2B, FA2B, FA2[3]G1, FA2[3]BG1, FA2G2, A2G2S1, A2G2S2, FA2G2S2, and FA2BG2S2). Subsequently, stepwise multivariate logistic regression was performed to further refine the selection of glycan peaks. Ultimately, FA2[3]G1, FA2G2, A2G2S2, and FA2BG2S2 were used to develop logistic regression models. We developed three logistic regression models to differentiate between the two types of sepsis. (1) Model 1 included FA2[3]G1, FA2G2, A2G2S2, and FA2BG2S2; (2) Model 2 included WBC, NEUT, AST, and ALT; (3) Model 3 included WBC, NEUT, AST, ALT, FA2[3]G1, FA2G2, A2G2S2, and FA2BG2S2.

### Validation and performance of the predictive model

**Figure 4:**
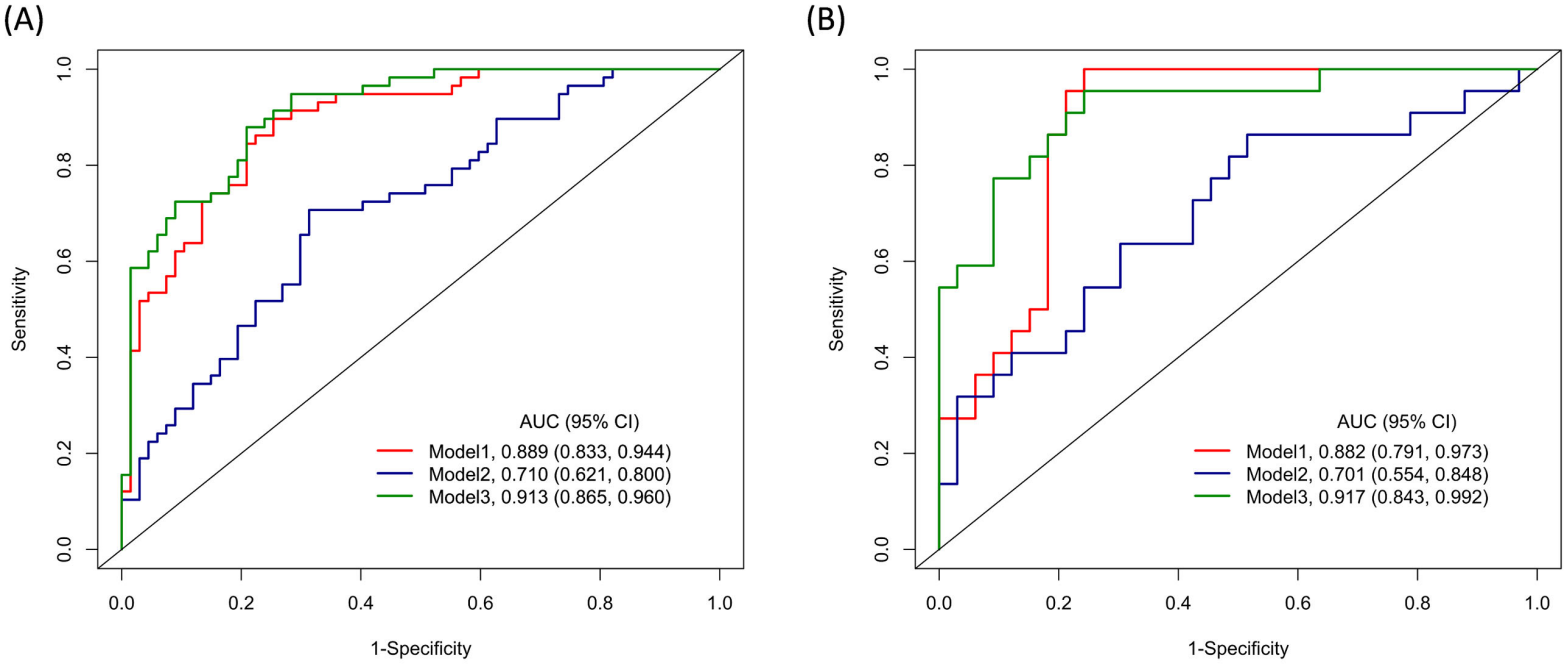
Receiver operating characteristic (ROC) curve analysis for glycan-based prediction models. Model 1 consists of FA2[3]G1, FA2G2, A2G2S2, and FA2BG2S2; Model 2 consists of WBC, NEUT, AST and ALT; Model 3 consists of WBC, NEUT, AST, ALT, FA2[3]G1, FA2G2, A2G2S2, and FA2BG2S2. **(A)** training set; **(B)** validation set. WBC, white blood cell; NEUT, absolute neutrophil count; ALT, alanine aminotransferase; AST, aspartate aminotransferase.

As illustrated in **Figure 4**, ROC curves were plotted to assess the performance of the three models. The AUC value of Model 1 was 0.889 (95% CI: 0.833, 0.944), which was significantly higher than Model 2 (AUC = 0.710, 95% CI: 0.621, 0.800). Notably, the AUC value of Model 3 was calculated to be 0.931 (95% CI: 0.865, 0.960), demonstrating enhanced predictive performance by integrating initial glycan peaks with conventional risk factors.

In the validation dataset, the three models achieved AUC values of 0.882 (95% CI: 0.791, 0.973), 0.701 (95% CI: 0.554, 0.848), and 0.917 (95% CI: 0.843, 0.992), respectively. Given its superior performance, Model 3 was chosen as the optimal predictive model for this study.

**Figure 5:**
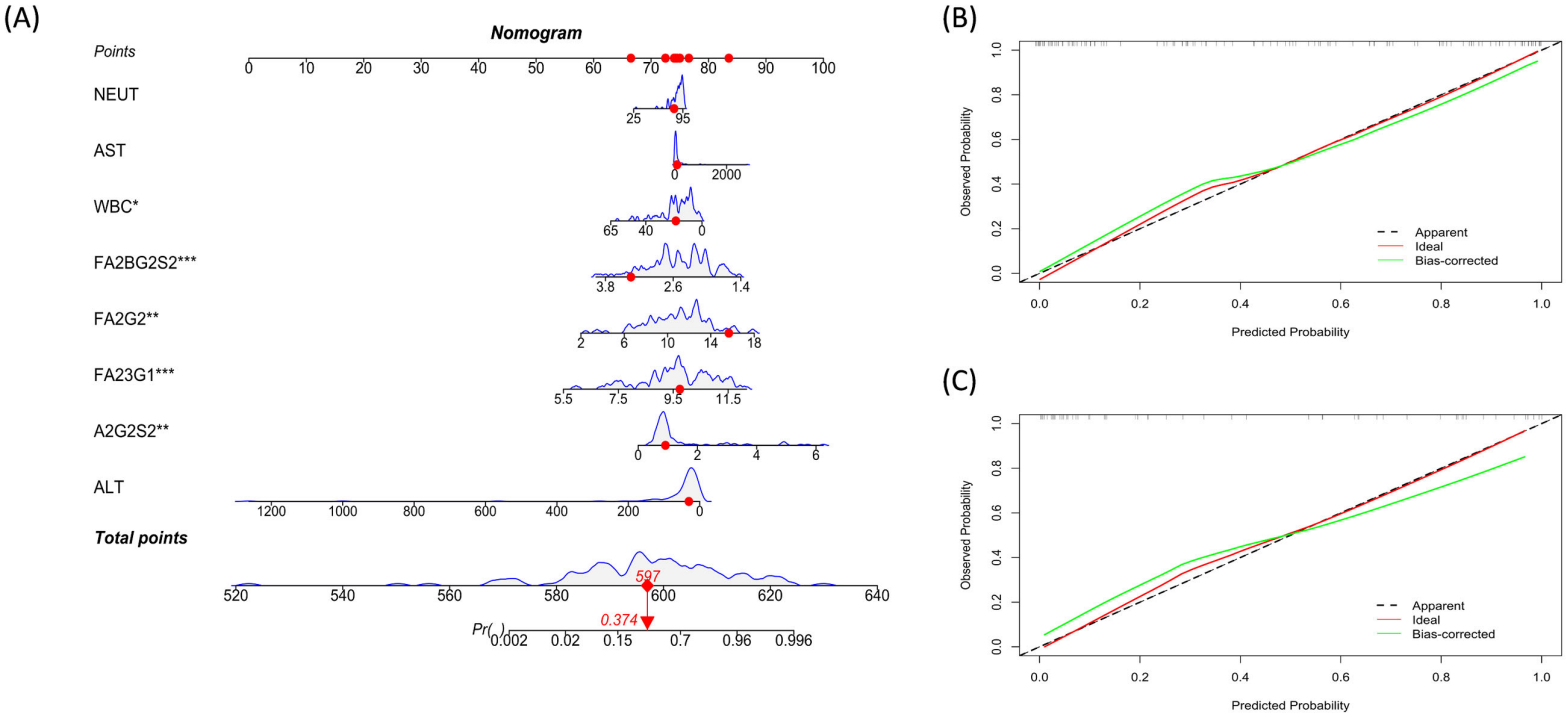
Nomogram and calibration curves for predicting the probability of sepsis type. **(A)** Nomogram for predicting sepsis type in patients caused by Gram-negative (0) and Gram-positive (1) bacteria; **(B)** Calibration plots in the training set; **(C)**Calibration plots in the validation set. Plot patient values on variable axes, draw vertical lines to the Points scale to determine partial scores, sum these for Total Points, and map this value to the bottom axis to read predicted sepsis type probability. B, bisecting N-acetylglucosamine (GlcNAc); F, fucose; G, galactose; S, Sialic acid. WBC, white blood cell; NEUT, absolute neutrophil count; ALT, alanine aminotransferase; AST, aspartate aminotransferase; GP, glycan peak.

A nomogram incorporating eight variables—including NEUT, AST, WBC, FA2BG2S2, FA2G2, FA2[3]G1, A2G2S2, and ALT—was constructed to differentiate between Gram-negative and Gram-positive sepsis (**Figure 5A**). The calibration curves demonstrated satisfactory agreement between predicted probabilities and observed outcomes in both the training set (**Figure 5B**) and validation set (**Figure 5C**). The Hosmer-Lemeshow test confirmed these findings, with nonsignificant goodness of fit results in the training set (*P* = 0.350) and validation set (*P* = 0.391).

### Differences in IgG glycome composition between sepsis survivors and non-survivors

As indicated in **Supplementary Tables 8**-**10**, 15 initial (FA1, A2, FA2, M5, FA2B, FA2[6]BG1, A2G2, A2BG2, FA2G2, FA2BG2, A2G2S1, FA2G2S1, FA2FG2S1, A2G2S2, and A2BG2S2) and 25 derived IgG *N*-glycan traits differed significantly between survivors and non-survivors. Regarding major glycosylation features, septic survivors exhibited significantly higher percentages of fucosylation (95.34%) and G0 (30.92%) than non-survivors, whereas sialylation (21.98%) and G2 (14.31%) were significantly lower. No significant differences were observed in bisecting GlcNAc and G1.

### The impact of IgG N-glycan peaks on 90-day mortality in septic patients

**Figure 6:**
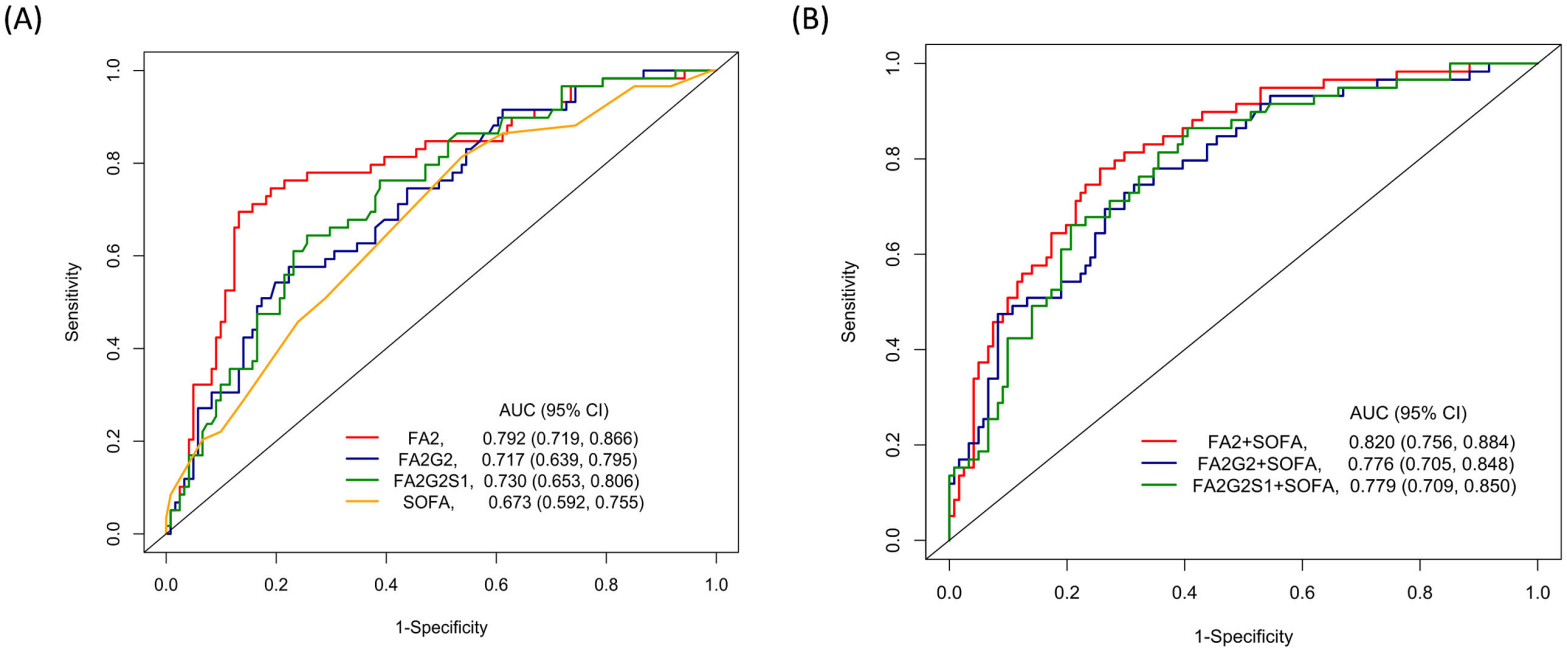
Discrimination capacity of initial glycan peaks and SOFA score for 90-day mortality among septic patients. **(a)** Efficacy of each indicator. **(b)** Efficacy of combined indicators. B, bisecting N-acetylglucosamine (GlcNAc); F, fucose; G, galactose; S, Sialic acid.

To evaluate the predictive performance of IgG *N*-glycans for 90-day mortality, we calculated the AUC, sensitivity (SE), specificity (SP), positive predictive value (PPV), and negative predictive value (NPV), with results presented in **Table 4** and **Figure 6A**. The AUC values were 0.792 (95% CI: 0.719, 0.866) for FA2, 0.717 (95% CI: 0.639, 0.795) for FA2G2, 0.730 (95% CI: 0.653, 0.806) for FA2G2S1, and 0.673 (95% CI: 0.592, 0.755) for SOFA. As shown in **Figure 6B**, combining SOFA with FA2, FA2G2, and FA2G2S1 achieved AUC values of 0.820 (95% CI: 0.756, 0.884), 0.776 (95% CI: 0.705, 0.848), and 0.779 (95% CI: 0.709, 0.850), respectively.

**Table 4:** SOFA and IgG *N*-glycans predicting 90-day mortality in sepsis.

Variable	AUC	95%LCI	95%UCI	Cut-off value	SE	SP	PPV	NPV
FA2	0.792	0.719	0.866	29.265	0.695	0.868	0.719	0.854
FA2G2	0.717	0.639	0.795	9.840	0.576	0.777	0.577	0.790
FA2G2S1	0.730	0.653	0.806	7.925	0.644	0.744	0.551	0.811
SOFA	0.673	0.592	0.755	7.500	0.814	0.463	0.425	0.836

AUC, Area Under the Curve; LCI, Lower Confidence interval; UCI, Upper Confidence interval; SE, sensitivity; SP, specificity; PPV, positive predictive value; NPV, negative predictive value; GP, glycan peak. SOFA, Sequential Organ Failure Assessment; F at the start of the abbreviation indicates a core-fucose α 1,6-linked to the inner GlcNAc; A2, biantennary with both GlcNAcs as *β* 1,2-linked; G (x), number (x) of *β* 1,4 linked galactose on antenna; F (x), number (x) of fucose linked α 1,3 to antenna GlcNAc; S (x), number (x) of sialic acids linked to galactoses.

### Independent predictors of 90-day mortality

As shown in **Table 5**, FA2 (OR: 1.188, 95% CI: 1.109, 1.273; *P* = 9.730E-07) and SOFA (OR: 1.234, 95% CI: 1.106, 1.378; *P* = 1.742E-04) independently predicted an increased risk of 90-day mortality. Conversely, FA2G2 (OR: 0.748, 95% CI: 0.658, 0.851; *P* = 1.020E-05) and FA2G2S1 (OR: 0.668, 95% CI: 0.561, 0.795; *P* = 5.722E-06) were associated with significantly lower mortality odds.

**Table 5:** The independent predictors of 90-day mortality.

Variable	*β*	SE	Walds	OR (95%CI)	*P*
FA2	0.173	0.035	23.981	1.188 (1.109, 1.273)	9.730E-07
FA2G2	-0.290	0.066	19.474	0.748 (0.658, 0.851)	1.020E-05
FA2G2S1	-0.404	0.089	20.579	0.668 (0.561, 0.795)	5.722E-06
SOFA	0.210	0.056	14.090	1.234 (1.106, 1.378)	1.742E-04

SOFA, Sequential Organ Failure Assessment; *β*, standardized regression coefficient; SE, Standard Error; OR, Odds Ratio; CI, Confidence Interval; F at the start of the abbreviation indicates a core-fucose α 1,6-linked to the inner GlcNAc; A2, biantennary with both GlcNAcs as *β* 1,2-linked; G (x), number (x) of *β* 1,4 linked galactose on antenna; F (x), number (x) of fucose linked α 1,3 to antenna GlcNAc; S (x), number (x) of sialic acids linked to galactoses. The concentrations of FA2, FA2G2, FA2G2S1, and SOFA were judged to be negative and positive according to the cut-off point value (FA2, 29.265; FA2G2, 9.840; FA2G2S1, 7.925; SOFA, 7.500).

### Comparisons of baseline characteristics and inflammatory cytokines in Gram-negative and Gram-positive sepsis patients relative to healthy controls

**Supplementary Tables 11** and **12** displayed the baseline characteristics of Gram-negative and Gram-positive sepsis patients versus healthy controls. There were no significant differences in Age and Gender. Gram-negative sepsis patients had significantly lower PLT than controls. Both patient groups exhibited higher WBC, NEUT, MPV, and AST levels than the healthy controls. In addition, the levels of inflammatory cytokines in Gram-negative and Gram-positive sepsis patients compared to the healthy control group are presented in **Supplementary Tables 13** and **14**. It is worth noting that the levels of IL-6 and IL-10 in both patient groups were markedly higher than those in the healthy control group.

### Comparisons of IgG glycome composition in Gram-negative and Gram-positive sepsis patients relative to healthy controls

As listed in **Supplementary Tables 15**-**17**, significant differences in 19 initial glycan peaks and 40 derived glycan traits were observed between patients with Gram-negative sepsis and healthy controls. For major glycosylation features, the levels of bisecting GlcNAc and G0 were significantly elevated in patients with Gram-negative sepsis relative to controls, whereas the levels of fucosylation, G1, and G2 were markedly reduced. Meanwhile, there were no significant findings regarding sialylation.

In addition, as depicted in **Supplementary Tables 18**-**20**, significant differences were observed in 15 initial glycan peaks and 35 derived glycan peaks between patients with Gram-positive sepsis and healthy controls. Similar to patients with Gram-negative sepsis, higher levels of bisecting GlcNAc and G0 were identified in patients with Gram-positive sepsis compared to the controls. Furthermore, the levels of sialylation, G1, and G2 were markedly reduced in patients with Gram-positive sepsis relative to the controls.

### Discrimination of Gram-negative and Gram-positive sepsis patients from healthy controls based on IgG N-glycosylation

As previously described, LASSO regression followed by stepwise logistic regression was conducted to screen for significant glycan peaks (**Supplementary Figures 6**, **7**). According to the screening results, M5, FA2G2, FA2FG2S1, and A2G2S2 were utilized for the diagnosis of patients with Gram-negative sepsis, while M5, A2G1, FA2[3]G1, and A2G2S1 were employed for diagnosing patients with Gram-positive sepsis. **Supplementary Figure 8** presented the ROC curve analysis, and the AUC values for the glycan-based models were 0.959 (95% CI: 0.922, 0.997) and 0.968 (95% CI: 0.941, 0.996), demonstrating outstanding diagnostic accuracy. Furthermore, in the validation dataset, the glycan-based models demonstrated AUCs of 0.951 (95% CI: 0.900, 1.000) and 0.931 (95% CI: 0.867, 0.995).

## Discussion

This nested case-control study comprehensively analyzed the IgG *N*-glycan profiles of patients with sepsis. We identified that multiple serum IgG *N*-glycans were altered in patients with Gram-negative sepsis relative to those with Gram-positive sepsis. The glycan peak-based predictive model we established holds potential for the early identification of pathogens responsible for sepsis. Furthermore, we observed that compared with non-survivors, septic patients who survived demonstrated significantly elevated sialylation and galactosylation levels, while fucosylation levels were significantly lower. IgG *N-*glycan peaks may serve as effective biomarkers for diagnosing and predicting the mortality risk in patients with sepsis. Additionally, we also found that the *N-*glycan compositions of both groups of sepsis patients significantly differed from those of healthy controls, which is consistent with previous findings in inflammatory diseases (17, 32, 33).

Sepsis is a systemic inflammatory response syndrome; due to its rapid progression, early pathogen detection and timely initiation of treatment are crucial for improving patient prognosis (34). Fucosylation of IgG inhibits the activation of antibody-dependent cellular cytotoxicity (ADCC) by reducing its binding to FcγRIIIa receptors on natural killer (NK) cells, thereby downregulating the pro-inflammatory activity of IgG (35). Galactosylation can enhance the affinity of IgG for the inhibitory receptor FccRIIb, resulting in increased anti-inflammatory effects (36, 37). As for sialylation, the spatial structure modified by sialylation affects the immunological activity of IgG (38). The absence of sialic acid significantly impacts the function of IgG, changing it from anti-inflammatory to pro-inflammatory (39, 40). In this study, we observed that patients with Gram-negative sepsis exhibited significantly lower fucosylation and galactosylation levels, as well as higher sialylation levels, compared to those with Gram-positive sepsis. This suggests that patients with Gram-negative sepsis have higher inflammatory levels, which aligns with earlier research findings (41, 42). In previous studies (43–45), inflammatory cytokines were used to differentiate between patients with Gram-positive and Gram-negative sepsis. However, the results of this study demonstrated no significant difference in inflammatory cytokines levels between the two groups. In contrast, IgG *N*-glycans exhibit superior discriminatory value.

In addition, we noted an association between inflammatory cytokines and glycan peaks (e.g., IL-6 and TNF-α), which further emphasizes the role of IgG *N*-glycosylation in regulating inflammatory diseases (46). Nomograms are a reliable and practical statistical tool that can quickly predict the probability of clinical events by integrating multiple variables and risk factors (47). The nomograms constructed in our study, based on glycan peak and conventional risk factors, effectively differentiate between patients with Gram-negative and Gram-positive sepsis, enabling rapid diagnosis and the initiation of appropriate treatment plans.

The high mortality rate of sepsis remains a persistent challenge in critical care medicine. Sepsis-induced injury is primarily mediated by an excessive inflammatory response (48). Previous studies have established a correlation between IgG glycosylation and clinical outcomes in several diseases (15, 35, 49). Furthermore, IgG glycosylation has been specifically linked to sepsis severity in another study (50). The SOFA score is widely used to quantify and predict the severity of organ dysfunction in sepsis (51, 52). In our study, several IgG *N*-glycans, particularly in fucosylation, G0, sialylation, and G2, were associated with survival outcomes. Our evaluation identified FA2 as the best independent predictor of 90-day mortality among IgG *N*-glycans, showing superior discriminatory power to the SOFA score (AUC 0.792 vs. 0.673). Patient outcomes in sepsis are closely linked to the severity of the inflammatory response. FA2 is known to enhance ADCC, potentially driving more severe inflammatory tissue damage (53). Therefore, we constructed combined models incorporating IgG *N-*glycans with the SOFA score and assessed their predictive performance. The combination of FA2 and SOFA enhanced predictive accuracy (AUC 0.820), suggesting a complementary role. The incremental value of this combination implies that IgG glycosylation may refine existing risk stratification models. These findings suggest that IgG *N*-glycans may serve as an excellent predictor of mortality risk in sepsis.

Although Gram-negative and Gram-positive sepsis are driven by distinct pathogenic mechanisms, the underlying differences that lead to divergent clinical prognoses have not been fully elucidated. The IgG *N*-glycans showed significant discriminatory power, revealing distinct patterns between Gram-negative and Gram-positive sepsis patients. Glycosylation markers have emerged as promising indicators with significant clinical utility.

Several limitations should be recognized in our study. First, our participants were recruited from Tianjin City in China. It is imperative that future research adopts a multicenter design to confirm and extend the present data. Second, our study is limited by a small sample size, a retrospective case-control design, and the lack of an independent external validation cohort; the 7:3 splitting was merely an internal resampling strategy and may overestimate generalizability. Therefore, larger prospective cohorts with external validation are warranted to confirm these results. Third, our study focused on distinguishing between the broad categories of Gram-negative and Gram-positive bacterial sepsis, rather than infections caused by specific pathogens. Fourth, variations in clinical severity and treatment strategies among patients constitute a confounding variable that may have influenced biomarker associations. The relationship between treatments and IgG *N*-glycosylation requires further investigation. Finally, we analyzed total serum IgG *N*-glycans rather than antigen-specific IgG. Antigen-specific IgG glycosylation differs from bulk IgG and changes dynamically during infection. However, given the highly heterogeneous and often polymicrobial nature of sepsis, isolating antigen-specific IgG for specific pathogens remains technically challenging. While total serum IgG may dilute some antigen-specific glycan signals, it provides a comprehensive reflection of the host’s systemic inflammatory and immunological state. Future studies focusing on antigen-specific IgG *N*-glycan variations are warranted to validate our findings and provide deeper mechanistic insights.

## Conclusion

Our study demonstrated that IgG *N*-glycosylation served as an effective discriminator between pathogenic Gram-negative and Gram-positive sepsis. Furthermore, a model combining IgG *N*-glycans with the SOFA score proved valuable for patient risk stratification. Given these findings, IgG *N*-glycosylation represents a promising biomarker to guide stratified treatment strategies in sepsis.

## Data Availability

The original contributions presented in the study are included in the article/**Supplementary Material**. Further inquiries can be directed to the corresponding authors.
