# Supplementary material for: Immunoglobulin G N-glycosylation predicts outcome in sepsis caused by pathogenic Gram-negative bacteria and Gram-positive bacteria: a nested case-control study
Source: Front Immunol. 2026 Jun 23;17:1826457. doi: 10.3389/fimmu.2026.1826457 (PMC13337443; doi:10.3389/fimmu.2026.1826457)

**Table S1** The calculation formula of IgG *N*-glycans

| **Glycan peaks** | **Description** | **Formula** |
| --- | --- | --- |
| ***Initially measured glycans*** |  |  |
| GP1 | Proportion of FA1 glycan in total IgG glycans | FA1 / GP * 100 |
| GP2 | Proportion of A2 glycan in total IgG glycans | A2 / GP * 100 |
| GP3 | Proportion of A2B glycan in total IgG glycans | A2B / GP * 100 |
| GP4 | Proportion of FA2 glycan in total IgG glycans | FA2 / GP * 100 |
| GP5 | Proportion of M5 glycan in total IgG glycans | M5 / GP * 100 |
| GP6 | Proportion of FA2B glycan in total IgG glycans | FA2B / GP * 100 |
| GP7 | Proportion of A2G1 glycan in total IgG glycans | A2G1 / GP * 100 |
| GP8 | Proportion of FA2[6]G1 glycan in total IgG glycans | FA2[6]G1 / GP * 100 |
| GP9 | Proportion of FA2[3]G1 glycan in total IgG glycans | FA2[3]G1 / GP * 100 |
| GP10 | Proportion of FA2[6]BG1 glycan in total IgG glycans | FA2[6]BG1 / GP * 100 |
| GP11 | Proportion of FA2[3]BG1 glycan in total IgG glycans | FA2[3]BG1 / GP * 100 |
| GP12 | Proportion of A2G2 glycan in total IgG glycans | A2G2 / GP * 100 |
| GP13 | Proportion of A2BG2 glycan in total IgG glycans | A2BG2 / GP * 100 |
| GP14 | Proportion of FA2G2 glycan in total IgG glycans | FA2G2 / GP * 100 |
| GP15 | Proportion of FA2BG2 glycan in total IgG glycans | FA2BG2 / GP * 100 |
| GP16 | Proportion of FA2G1S1 glycan in total IgG glycans | FA2G1S1 / GP * 100 |
| GP17 | Proportion of A2G2S1 glycan in total IgG glycans | A2G2S1 / GP * 100 |
| GP18 | Proportion of FA2G2S1 glycan in total IgG glycans | FA2G2S1 / GP * 100 |
| GP19 | Proportion of FA2BG2S1 glycan in total IgG glycans | FA2BG2S1 / GP * 100 |
| GP20 | Proportion of FA2FG2S1 glycan in total IgG glycans | FA2FG2S1 / GP * 100 |
| GP21 | Proportion of A2G2S2 glycan in total IgG glycans | A2G2S2 / GP * 100 |
| GP22 | Proportion of A2BG2S2 glycan in total IgG glycans | A2BG2S2 / GP * 100 |
| GP23 | Proportion of FA2G2S2 glycan in total IgG glycans | FA2G2S2 / GP * 100 |
| GP24 | Proportion of FA2BG2S2 glycan in total IgG glycans | FA2BG2S2 / GP * 100 |
| ***Derived glycosylation traits*** |  |  |
| **Sialylated glycans** |  |  |
| FGS/(FG+FGS) | Proportion of sialylation of fucosylated galactosylated glycans without bisecting GlcNAc in total IgG glycans | SUM (FA2G1S1 + FA2G2S1 + FA2G2S2) /  SUM (FA2G1S1 + FA2G2S1 + FA2G2S2 +  FA2[6]G1 + FA2[3]G1 + FA2G2) * 100 |
| FBGS/(FBG+FBGS) | Proportion of sialylation of fucosylated galactosylated glycans with bisecting GlcNAc in total IgG glycans | SUM (FA2BG2S1 + FA2BG2S2) /  SUM (FA2BG2S1 + FA2BG2S2 + FA2[6]BG1 +  FA2[3]BG1 + FA2BG2) * 100 |
| FGS/(F+FG+FGS) | Proportion of sialylation of all fucosylated glycans without bisecting GlcNAc in total IgG glycans | SUM (FA2G1S1 + FA2G2S1 + FA2G2S2) /  SUM (FA2G1S1 + FA2G2S1 + FA2G2S2 +  FA2 + FA2[6]G1 + FA2[3]G1 + FA2G2) *  100 |
| FBGS/(FB+FBG+FBGS) | Proportion of sialylation of all fucosylated glycans with bisecting GlcNAc in total IgG glycans | SUM (FA2BG2S1 + FA2BG2S2) /  SUM (FA2BG2S1 + FA2BG2S2 + FA2B +  FA2[6]BG1 + FA2[3]BG1 + FA2BG2) * 100 |
| FG1S1/(FG1+FG1S1) | Proportion of monosialylation of fucosylated monogalactosylated glycans without bisecting GlcNAc in total IgG glycans | FA2G1S1 / SUM (FA2G1S1 + FA2[6]G1 +  FA2[3]G1) * 100 |
| FG2S1/(FG2+FG2S1+FG2S2) | Proportion of monosialylation of fucosylated digalactosylated glycans without bisecting GlcNAc in total IgG glycans | FA2G2S1 / SUM (FA2G2S1 + FA2G2 +  FA2G2S2) * 100 |
| FG2S2/(FG2+FG2S1+FG2S2) | Proportion of disialylation of fucosylated digalactosylated glycans without bisecting GlcNAc in total IgG glycans | FA2G2S2 / SUM (FA2G2S2 + FA2G2 +  FA2G2S1) * 100 |
| FBG2S1/(FBG2+FBG2S1+FBG2S2) | Proportion of monosialylation of fucosylated digalactosylated glycans with bisecting GlcNAc in total IgG glycans | FA2BG2S1 / SUM (FA2BG2S1 + FA2BG2 +  FA2BG2S2) * 100 |
| FBG2S2/(FBG2+FBG2S1+FBG2S2) | Proportion of disialylation of fucosylated digalactosylated glycans with bisecting GlcNAc in total IgG glycans | FA2BG2S2 / SUM (FA2BG2S2 + FA2BG2 +  FA2BG2S1) * 100 |
| F^total^S1/F^total^S2 | Proportion of all fucosylated monosialylated and disialylated glycans (+/- bisecting GlyNAc) in total IgG glycans | SUM (FA2G1S1 + FA2G2S1 + FA2BG2S1) /  SUM (FA2G2S2 + FA2BG2S2) |
| FS1/FS2 | Proportion of fucosylated monosialylated and disialylated glycans (without bisecting GlcNAc) in total IgG glycans | SUM (FA2G1S1 + FA2G2S1) / FA2G2S2 |
| FBS1/FBS2 | Proportion of fucosylated monosialylated and disialylated glycans (with bisecting GlcNAc) in total IgG glycans | FA2BG2S1 / FA2BG2S2 |
| **Bisecting *N*-acetylglucosamine glycans** |  |  |
| FBS^total^/FS^total^ | Proportion of all fucosylated sialylated glycans with and without bisecting GlcNAc in total IgG glycans | SUM (FA2BG2S1 + FA2BG2S2) /  SUM (FA2G1S1 + FA2G2S1 + FA2G2S2) |
| FBS1/FS1 | Proportion of fucosylated monosialylated glycans with and without bisecting GlcNAc in total IgG glycans | FA2BG2S1 / SUM (FA2G1S1 + FA2G2S1) |
| FBS1/(FS1+FBS1) | The incidence of bisecting GlcNAc in all fucosylated monosialylated glycans in total IgG glycans in total IgG glycans | FA2BG2S1 / SUM (FA2G1S1 + FA2G2S1 +  FA2BG2S1) |
| FBS2/FS2 | Proportion of fucosylated disialylated glycans with and without bisecting GlcNAc in total IgG glycans | FA2BG2S2 / FA2G2S2 |
| FBS2/(FS2+FBS2) | The incidence of bisecting GlcNAc in all fucosylated disialylated glycans in total IgG glycans | FA2BG2S2 / SUM (FA2G2S2 + FA2BG2S2) |
| **Neutral glycans** |  |  |
| FA1^n^ | Proportion of FA1 glycan in total neutral IgG glycans (GPn) | FA1 / GP^n^ * 100 |
| A2^n^ | Proportion of A2 glycan in total neutral IgG glycans (GPn) | A2 / GP^n^ * 100 |
| FA2^n^ | Proportion of FA2 glycan in total neutral IgG glycans (GPn) | FA2 / GP^n^ * 100 |
| M5^n^ | Proportion of M5 glycan in total neutral IgG glycans (GPn) | M5 / GP^n^ * 100 |
| FA2B^n^ | Proportion of FA2B glycan in total neutral IgG glycans (GPn) | FA2B / GP^n^ * 100 |
| A2G1^n^ | Proportion of A2G1 glycan in total neutral IgG glycans (GPn) | A2G1 / GP^n^ * 100 |
| FA2[6]G1^n^ | Proportion of FA2[6]G1 glycan in total neutral IgG glycans (GPn) | FA2[6]G1 / GP^n^ * 100 |
| FA2[3]G1^n^ | Proportion of FA2[3]G1 glycan in total neutral IgG glycans (GPn) | FA2[3]G1 / GP^n^ * 100 |
| FA2[6]BG1^n^ | Proportion of FA2[6]BG1 glycan in total neutral IgG glycans (GPn) | FA2[6]BG1 / GP^n^ * 100 |
| FA2[3]BG1^n^ | Proportion of FA2[3]BG1 glycan in total neutral IgG glycans (GPn) | FA2[3]BG1 / GP^n^ * 100 |
| A2G2^n^ | Proportion of A2G2 glycan in total neutral IgG glycans (GPn) | A2G2 / GP^n^ * 100 |
| A2BG2 ^n^ | Proportion of A2BG2 glycan in total neutral IgG glycans (GPn) | A2BG2 / GP^n^ * 100 |
| FA2G2 ^n^ | Proportion of FA2G2 glycan in total neutral IgG glycans (GPn) | FA2G2 / GP^n^ * 100 |
| FA2BG2^n^ | Proportion of FA2BG2 glycan in total neutral IgG glycans (GPn) | FA2BG2 / GP^n^ * 100 |
| **Galactosylated glycans** |  |  |
| G0 | Proportion of agalactosylated glycans in total neutral IgG glycans | SUM (FA1^n^: FA2^n^ + FA2B^n^) |
| G1 | Proportion of monogalactosylated glycans in total neutral IgG glycans | SUM (A2G1^n^: FA2[3]BG1^n^) |
| G2 | Proportion of digalactosylated glycans in total neutral IgG glycans | SUM (A2G2^n^: FA2BG2^n^) |
| **Core fucosylated glycans** |  |  |
| F^n^ ^total^ | The sum of all fucosylated glycans (+/- bisecting GlcNAc) in total neutral IgG glycans | SUM (FA1^n^+ FA2^n^+ FA2B^n^+ FA2[6]G1^n^+ FA2[3]G1^n^+ P10^n^+ FA2[3]BG1^n^+ FA2G2 ^n^+ FA2BG2^n^) |
| FG0 ^total^/G0 | Proportion of fucosylation of agalactosylated glycans in total neutral IgG glycans | SUM (FA1^n^+ FA2^n^+ FA2B^n^) / G0 * 100 |
| FG1 ^total^/G1 | Proportion of fucosylation of monogalactosylated glycans in total neutral IgG glycans | SUM (FA2[6]G1^n^+ FA2[3]G1^n^+ FA2[6]BG1^n^+ FA2[3]BG1^n^) / G1 * 100 |
| FG2 ^total^/G2 | Proportion of fucosylation of digalactosylated glycans in total neutral IgG glycans | SUM (FA2G2 ^n^+ FA2BG2) / G2 * 100 |
| F^n^ | The sum of fucosylated glycans (without bisecting GlcNAc) in total neutral IgG glycans | SUM (FA1^n^+ FA2^n^+ FA2[6]G1^n^+ FA2[3]G1^n^+ FA2G2 ^n^) |
| FG0/G0 | Proportion of fucosylation of agalactosylated glycans (without bisecting GlcNAc) in total neutral IgG glycans | SUM (FA1^n^+ FA2^n^) / G0 * 100 |
| FG1/G1 | Proportion of fucosylation of monogalactosylated glycans (without bisecting GlcNAc) in total neutral IgG glycans | SUM (FA2[6]G1^n^+ FA2[3]G1^n^) / G1 * 100 |
| FG2/G2 | Proportion of fucosylation of digalactosylated glycans (without bisecting GlcNAc) in total neutral IgG glycans | FA2G2 ^n^ / G2 * 100 |
| **Core fucosylation and bisecting *N*-acetylglucosamine** |  |  |
| FB^n^ | The sum of fucosylated glycans (with bisecting GlcNAc) in total neutral IgG glycans | SUM (FA2B^n^ + FA2[6]BG1^n^ + FA2[3]BG1^n^ + FA2BG2^n^) |
| FBG0/G0 | Proportion of fucosylation of agalactosylated glycans (with bisecting GlcNAc) in total neutral IgG glycans | FA2B^n^ / G0 * 100 |
| FBG1/G1 | Proportion of fucosylation of monogalactosylated glycans (with bisecting GlcNAc) in total neutral IgG glycans | SUM (FA2[6]BG1^n^ + FA2[3]BG1^n^) / G1 * 100 |
| FBG2/G2 | Proportion of fucosylation of digalactosylated glycans (with bisecting GlcNAc) in total neutral IgG glycans | FA2BG2^n^ / G2 * 100 |
| FB^n^/F^n^ | Proportion of fucosylated glycans with and without bisecting GlcNAc in total neutral IgG glycans | FB^n^/ F^n^ * 100 |
| FB^n^/F^n total^ | The incidence of bisecting GlcNAc in all fucosylated glycans in total neutral IgG glycans | FB^n^/ F^n total^ * 100 |
| F^n^/(B^n^+FB^n^) | Proportion of fucosylated non-bisecting GlcNAc glycans and all glycans with bisecting GlcNAc in total neutral IgG glycans | F^n^/ (A2BG2 ^n^ + FB^n^) |
| B^n^/(F^n^+FB^n^) | Proportion of glycans with bisecting GlcNAc and all fucosylated glycans (+/- bisecting GlcNAc) in total neutral IgG glycans | A2BG2 ^n^/ (F^n^+ FB^n^) * 1000 |
| FBG2/FG2 | Proportion of fucosylated digalactosylated glycans with and without bisecting GlcNAc in total neutral IgG glycans | FA2BG2^n^/FA2G2 ^n^ |
| FBG2/(FG2+FBG2) | The incidence of bisecting GlcNAc in all fucosylated digalactosylated glycans in total neutral IgG glycans | FA2BG2^n^/ (FA2G2 ^n^ + FA2BG2^n^) * 100 |
| FG2/(BG2+FBG2) | Proportion of fucosylated digalactosylated non-bisecting GlcNAc glycans and all digalactosylated glycans with bisecting GlcNAc in total neutral IgG glycans | FA2G2 ^n^/ (A2BG2 ^n^ + FA2BG2^n^) |
| BG2/(FG2+FBG2) | Proportion of digalactosylated glycans with bisecting GlcNAc and all fucosylated digalactosylated glycans (+/- bisecting GlcNAc) in total neutral IgG glycans | A2BG2 ^n^/ (FA2G2 ^n^ + FA2BG2^n^) * 1000 |
| **Four major glycosylation features** |  |  |
| Fucosylation | Proportion of fucosylated glycans in total IgG glycans | FA1+ FA2+ FA2B+ FA2[6]G1+ FA2[3]G1+ FA2[6]BG1+ FA2[3]BG1+ FA2G2 + FA2BG2+ FA2G1S1+ FA2G2S1+ FA2BG2S1+ FA2G2S2+ FA2BG2S2 |
| Bisecting GlcNAc | Proportion of bisecting glycans in total IgG glycans | A2B+ FA2B+ FA2[6]BG1+ FA2[3]BG1+ A2BG2 + FA2BG2+ FA2BG2S1+ A2BG2S2 + FA2BG2S2 |
| Sialylation | Proportion of sialylated glycans in total IgG glycans | FA2G1S1+A2G2S1 +FA2G2S1+FA2BG2S1+A2G2S2+A2BG2S2 +FA2G2S2+FA2BG2S2 |
| Galactosylation |  |  |
| G0 | Proportion of agalactosylated glycans in total IgG glycans | FA1+ A2+ A2B+ FA2+ FA2B |
| G1 | Proportion of monogalactosylated glycans in total IgG glycans | A2G1+ FA2[6]G1+ FA2[3]G1+ FA2[6]BG1+ FA2[3]BG1 |
| G2 | Proportion of galactosylated glycans in total IgG glycans | A2G2+ A2BG2 + FA2G2 + FA2BG2 |

Initial glycans compositions: F at the start of the abbreviation indicates a core-fucose α 1,6-linked to the inner GlcNAc; Mx, number (x) of mannose on core GlcNAcs; Ax, number of antenna (GlcNAc) on trimannosyl core; A2, biantennary with both GlcNAcs as *β* 1,2-linked; B, bisecting GlcNAc linked β1,4 to β 1,3 mannose; G (x), number (x) of *β* 1,4 linked galactose on antenna; F (x), number (x) of fucose linked α 1,3 to antenna GlcNAc; S (x), number (x) of sialic acids linked to galactoses. GlcNAc, *N-*acetylglucosamine; GP, glycan peak; G0, agalactosylation; G1, monogalactosylation; G2, digalactosylation; IgG, immunoglobulin G.

Table S2 Levels of circulating inflammatory cytokines in patients with sepsis

| Inflammatory cytokines | Patients with Gram-negative sepsis  (n = 100) | Patients with Gram-  positive sepsis  (n = 80) | *P* | *q* |
| --- | --- | --- | --- | --- |
| IL-2 (ng/L) | 1.02 (0.53, 1.58) | 0.64 (0.31, 1.35) | 0.065 | 0.195 |
| IL-4 (ng/L) | 0.45 (0.00, 1.05) | 0.00 (0.00, 0.96) | 0.039 | 0.195 |
| IL-6 (ng/L) | 589.15(217.28, 3418.69) | 359.74 (132.75, 1960.59) | 0.189 | 0.378 |
| IL-10 (ng/L) | 24.24 (5.71, 39.25) | 16.06 (5.78, 37.62) | 0.652 | 0.808 |
| TNF-α(ng/L) | 0.00 (0.00, 0.58) | 0.00 (0.00, 0.90) | 0.673 | 0.808 |
| IFN-γ(ng/L) | 0.84 (0.00, 3.52) | 0.57 (0.00, 3.42) | 0.926 | 0.926 |

FDR, false discovery rate; IL-2, interleukin-2; IL-4, interleukin-4; IL-6, interleukin-6; IL-10, interleukin-10; TNF-α, tumor necrosis factor-α. IFN-γ, interferon-γ; *P* < 0.05 was considered statistically significant; *q* < 0.05 was considered statistically significant after correction using FDR.

Table S3 Comparisons of the 54 derived glycans between patients with sepsis

| Derived glycosylation traits | Patients with Gram-negative sepsis  (n = 100) | Patients with Gram-positive sepsis  (n = 80) | *P* | *q* |
| --- | --- | --- | --- | --- |
| **Sialylated glycans** |  |  |  |  |
| FGS/(FG+FGS) | 26.06 (23.98, 28.51) | 26.92 (24.78, 28.77) | 0.175 | 0.214 |
| FBGS/(FBG+FBGS) | 42.98 (39.52, 46.09) | 41.09 (36.79, 44.33) | 0.036 | 0.059 |
| FGS/(F+FG+FGS) | 16.67 (14.30, 18.75) | 18.09 (16.47, 20.21) | 0.005 | 0.012 |
| FBGS/(FB+FBG+FBGS) | 28.35 (25.24, 31.52) | 28.76 (24.97, 32.49) | 0.581 | 0.592 |
| FG1S1/(FG1+FG1S1) | 10.79 (9.65, 11.92) | 10.47 (9.30, 11.63) | 0.114 | 0.151 |
| FG2S1/(FG2+FG2S1+FG2S2) | 38.97 (37.55, 41.19) | 40.15 (37.95, 41.74) | 0.140 | 0.176 |
| FG2S2/(FG2+FG2S1+FG2S2) | 10.02 (9.02, 12.21) | 8.06 (7.21, 9.32) | 2.746E-09 | 2.472E-08 |
| FBG2S1/(FBG2+FBG2S1+FBG2S2) | 35.73 (32.95, 38.04) | 36.94 (33.89, 40.13) | 0.030 | 0.051 |
| FBG2S2/(FBG2+FBG2S1+FBG2S2) | 40.99 (37.96, 43.62) | 40.31 (37.15, 43.41) | 0.431 | 0.475 |
| F^total^S1/F^total^S2 | 2.84 (2.58, 3.24) | 3.36 (3.14, 3.66) | 7.866E-10 | 8.495E-09 |
| FS1/FS2 | 5.44 (4.82, 6.01) | 6.43 (5.87, 7.26) | 6.993E-07 | 4.387E-06 |
| FBS1/FBS2 | 0.88 (0.79, 0.98) | 0.91 (0.82, 1.03) | 0.089 | 0.123 |
| **Bisecting *N-*acetylglucosamine glycans** |  |  |  |  |
| FBS^total^/FS^total^ | 0.37 (0.33, 0.44) | 0.33 (0.29, 0.37) | 3.371E-05 | 1.138E-04 |
| FBS1/FS1 | 0.20 (0.18, 0.25) | 0.18 (0.15, 0.22) | 1.832E-03 | 4.300E-03 |
| FBS1/(FS1+FBS1) | 0.17 (0.15, 0.20) | 0.15 (0.13, 0.18) | 1.832E-03 | 4.300E-03 |
| FBS2/FS2 | 1.28 (1.09, 1.51) | 1.26 (1.13, 1.41) | 0.488 | 0.507 |
| FBS2/(FS2+FBS2) | 0.56 (0.52, 0.60) | 0.56 (0.53, 0.59) | 0.487 | 0.507 |
| **Neutral glycans** |  |  |  |  |
| FA1^n^ | 0.44 (0.30, 0.65) | 0.31 (0.24, 0.39) | 1.018E-06 | 4.997E-06 |
| A2^n^ | 1.06 (0.66, 1.59) | 0.74 (0.47, 1.10) | 7.144E-04 | 1.929E-03 |
| FA2^n^ | 34.27 (29.38, 40.05) | 31.76 (29.47, 34.41) | 8.839E-03 | 0.018 |
| M5^n^ | 0.13 (0.10, 0.17) | 0.20 (0.13, 0.26) | 7.311E-07 | 4.387E-06 |
| FA2B^n^ | 7.11 (5.94, 7.94) | 5.95 (5.32, 6.51) | 8.727E-07 | 4.712E-06 |
| A2G1^n^ | 0.55 (0.40, 0.74) | 0.72 (0.53, 0.95) | 6.895E-05 | 2.190E-04 |
| FA2[6]G1^n^ | 21.77 (20.28, 23.40) | 21.26 (19.91, 22.85) | 0.333 | 0.374 |
| FA2[3]G1^n^ | 11.79 (9.98, 13.10) | 12.81 (12.02, 13.83) | 4.810E-06 | 1.998E-05 |
| FA2[6]BG1^n^ | 5.48 (4.67, 6.07) | 5.55 (5.04, 6.31) | 0.252 | 0.296 |
| FA2[3]BG1^n^ | 0.98 (0.87, 1.14) | 1.20 (0.96, 1.34) | 2.333E-05 | 8.400E-05 |
| A2G2^n^ | 0.68 (0.47, 1.02) | 0.94 (0.72, 1.23) | 1.560E-04 | 4.435E-04 |
| A2BG2 ^n^ | 0.36 (0.30, 0.45) | 0.42 (0.30, 0.54) | 0.014 | 0.025 |
| FA2G2 ^n^ | 12.29 (9.53, 15.36) | 15.19 (13.58, 16.79) | 2.231E-06 | 1.004E-05 |
| FA2BG2^n^ | 1.77 (1.61, 2.03) | 1.67 (1.31, 1.92) | 0.011 | 0.020 |
| **Galactosylated glycans** |  |  |  |  |
| G0^n^ | 43.12 (37.21, 49.26) | 38.87 (36.35, 41.99) | 8.982E-05 | 2.695E-04 |
| G1^n^ | 40.82 (37.71, 42.96) | 41.83 (40.71, 43.66) | 9.065E-03 | 0.018 |
| G2^n^ | 15.35 (12.14, 18.29) | 18.45 (16.18, 19.86) | 6.152E-06 | 2.373E-05 |
| **Core fucosylated glycans** |  |  |  |  |
| F^n total^ | 96.71 (95.72, 97.56) | 96.55 (95.84, 97.18) | 0.121 | 0.156 |
| FG0^n total^/G0^n^ | 97.48 (96.34, 98.45) | 97.97 (97.19, 98.74) | 8.617E-03 | 0.018 |
| FG1^n total^/G1^n^ | 98.62 (98.18, 99.02) | 98.28 (97.72, 98.75) | 1.403E-03 | 3.608E-03 |
| FG2^n total^ /G2^n^ | 92.58 (90.94, 94.35) | 92.48 (91.08, 93.63) | 0.688 | 0.688 |
| F^n^ | 81.92 (79.56, 83.30) | 82.03 (80.53, 83.22) | 0.306 | 0.352 |
| FG0^n^/G0^n^ | 81.59 (77.87, 83.31) | 82.81 (80.08, 85.01) | 9.857E-03 | 0.019 |
| FG1^n^/G1^n^ | 82.84 (80.93, 84.51) | 81.97 (80.94, 83.28) | 0.071 | 0.101 |
| FG2^n^/G2^n^ | 80.59 (77.48, 82.57) | 83.37 (81.35, 85.29) | 1.304E-07 | 1.006E-06 |
| **Core fucosylation and bisecting *N-*acetylglucosamine** |  |  |  |  |
| FB^n^ | 14.98 (13.56, 16.98) | 14.43 (13.48, 15.77) | 0.054 | 0.084 |
| FBG0^n^/G0^n^ | 16.16 (14.07, 18.88) | 15.20 (14.01, 16.76) | 0.042 | 0.066 |
| FBG1^n^/G1^n^ | 15.59 (14.19, 17.85) | 16.30 (14.86, 17.60) | 0.441 | 0.477 |
| FBG2^n^/G2^n^ | 12.12 (10.57, 13.83) | 8.99 (7.71, 10.08) | 3.707E-17 | 1.531E-15 |
| FB^n^/F^n^ | 18.58 (16.23, 21.27) | 17.54 (16.31, 19.46) | 0.064 | 0.094 |
| FB^n^/F^n total^ | 15.67 (13.97, 17.54) | 14.92 (14.03, 16.29) | 0.064 | 0.094 |
| F^n^/(B^n^ + FB^n^) | 5.23 (4.58, 6.00) | 5.53 (5.02, 5.86) | 0.094 | 0.127 |
| B^n^/(F^n^ + FB^n^) | 3.71 (3.06, 4.70) | 4.41 (3.11, 5.56) | 0.016 | 0.028 |
| FBG2^n^/FG2^n^ | 0.15 (0.13, 0.18) | 0.11 (0.09, 0.12) | 8.508E-17 | 1.531E-15 |
| FBG2^n^ /(FG2^n^ + FBG2^n^) | 13.16 (11.29, 15.29) | 9.97 (8.39, 10.92) | 8.508E-17 | 1.531E-15 |
| FG2^n^/(BG2^n^ + FBG2^n^) | 5.50 (4.68, 6.41) | 7.17 (6.39, 8.54) | 4.546E-15 | 6.137E-14 |
| BG2^n^/(FG2^n^ + FBG2^n^) | 26.03 (20.42, 33.64) | 25.11 (18.67, 31.23) | 0.238 | 0.286 |

B, bisecting *N*-acetylglucosamine (GlcNAc); F, fucose; FDR, false discovery rate; G, galactose; GP, glycan peak; S, Sialic acid. *P* < 0.05 was considered statistically significant; *q* < 0.05 was considered statistically significant after correction using FDR.

Table S4 Univariate logistic regression analysis of 24 initial glycans

| Glycan peaks | β | SE | OR (95% CI) | *P* | *q* |
| --- | --- | --- | --- | --- | --- |
| FA1 | -0.615 | 0.145 | 0.541 (0.403, 0.713) | 2.202E-05 | 1.608E-04 |
| A2 | -0.491 | 0.141 | 0.612 (0.461, 0.803) | 5.115E-04 | 1.116E-03 |
| A2B | 0.582 | 0.143 | 1.791 (1.362, 2.390) | 4.647E-05 | 1.608E-04 |
| FA2 | -0.322 | 0.138 | 0.725 (0.551, 0.947) | 1.956E-02 | 2.934E-02 |
| M5 | 0.809 | 0.149 | 2.245 (1.690, 3.047) | 6.576E-08 | 1.578E-06 |
| FA2B | -0.603 | 0.147 | 0.547 (0.406, 0.724) | 4.021E-05 | 1.608E-04 |
| A2G1 | 0.524 | 0.142 | 1.689 (1.285, 2.251) | 2.319E-04 | 5.566E-04 |
| FA2[6]G1 | -0.179 | 0.134 | 0.835 (0.640, 1.085) | 1.799E-01 | 2.159E-01 |
| FA2[3]G1 | 0.666 | 0.149 | 1.946 (1.463, 2.635) | 8.435E-06 | 5.061E-05 |
| FA2[6]BG1 | 0.189 | 0.135 | 1.208 (0.927, 1.580) | 1.624E-01 | 2.051E-01 |
| FA2[3]BG1 | 0.649 | 0.151 | 1.913 (1.435, 2.596) | 1.666E-05 | 7.997E-05 |
| A2G2 | 0.554 | 0.143 | 1.739 (1.321, 2.323) | 1.136E-04 | 3.029E-04 |
| A2BG2 | 0.308 | 0.134 | 1.360 (1.048, 1.778) | 2.216E-02 | 3.128E-02 |
| FA2G2 | 0.701 | 0.151 | 2.017 (1.512, 2.742) | 3.544E-06 | 2.835E-05 |
| FA2BG2 | -0.412 | 0.14 | 0.662 (0.499, 0.867) | 3.293E-03 | 5.645E-03 |
| FA2G1S1 | -0.074 | 0.134 | 0.928 (0.712, 1.208) | 5.814E-01 | 5.814E-01 |
| A2G2S1 | 0.473 | 0.142 | 1.605 (1.220, 2.136) | 8.913E-04 | 1.783E-03 |
| FA2G2S1 | 0.729 | 0.153 | 2.073 (1.549 ,2.831) | 1.953E-06 | 2.344E-05 |
| FA2BG2S1 | -0.091 | 0.134 | 0.912 (0.701, 1.186) | 4.951E-01 | 5.166E-01 |
| FA2FG2S1 | -0.452 | 0.141 | 0.636 (0.479, 0.834) | 1.315E-03 | 2.428E-03 |
| A2G2S2 | 0.347 | 0.138 | 1.415 (1.082, 1.864) | 1.213E-02 | 1.941E-02 |
| A2BG2S2 | 0.211 | 0.131 | 1.235 (0.956, 1.602) | 1.076E-01 | 1.435E-01 |
| FA2G2S2 | -0.118 | 0.135 | 0.888 (0.681, 1.156) | 3.797E-01 | 4.142E-01 |
| FA2BG2S2 | -0.159 | 0.135 | 0.852 (0.653, 1.108) | 2.358E-01 | 2.695E-01 |

Initial glycans compositions: F at the start of the abbreviation indicates a core-fucose α 1,6-linked to the inner GlcNAc; Mx, number (x) of mannose on core GlcNAcs; Ax, number of antenna (GlcNAc) on trimannosyl core; A2, biantennary with both GlcNAcs as *β* 1,2-linked; B, bisecting GlcNAc linked β1,4 to β 1,3 mannose; G (x), number (x) of *β* 1,4 linked galactose on antenna; F (x), number (x) of fucose linked α 1,3 to antenna GlcNAc; S (x), number (x) of sialic acids linked to galactoses.CI, confidence interval; FDR, false discovery rate; GP, glycan peak; OR, odds ratio; SE, standard error; *β*, regression coefficient. *P* < 0.05 was considered statistically significant, *q* < 0.05 was considered statistically significant after correction using FDR

Table S5 Multivariate logistic regression analysis of 24 initial glycans

| Glycan peaks | β | SE | OR (95%CI) | *P* | *q* |
| --- | --- | --- | --- | --- | --- |
| FA1 | -0.68 | 0.162 | 0.506(0.364, 0.690) | 2.804E-05 | 6.810E-05 |
| A2 | -0.508 | 0.151 | 0.602(0.443, 0.805) | 7.964E-04 | 1.231E-03 |
| A2B | 0.629 | 0.159 | 1.875(1.385, 2.589) | 7.640E-05 | 1.624E-04 |
| FA2 | -0.353 | 0.15 | 0.702(0.520, 0.939) | 1.872E-02 | 2.122E-02 |
| M5 | 0.865 | 0.166 | 2.374(1.737, 3.334) | 1.764E-07 | 2.999E-06 |
| FA2B | -0.693 | 0.165 | 0.500(0.357, 0.684) | 2.622E-05 | 6.810E-05 |
| A2G1 | 0.519 | 0.152 | 1.680(1.254, 2.283) | 6.638E-04 | 1.128E-03 |
| FA2[3]G1 | 0.845 | 0.172 | 2.328(1.682, 3.315) | 9.332E-07 | 7.932E-06 |
| FA2[3]BG1 | 0.739 | 0.165 | 2.094(1.530, 2.935) | 7.937E-06 | 2.699E-05 |
| A2G2 | 0.526 | 0.154 | 1.692(1.259, 2.310) | 6.463E-04 | 1.128E-03 |
| A2BG2 | 0.295 | 0.144 | 1.343(1.017, 1.788) | 3.992E-02 | 3.992E-02 |
| FA2G2 | 0.773 | 0.166 | 2.167(1.582, 3.044) | 3.276E-06 | 1.392E-05 |
| FA2BG2 | -0.356 | 0.148 | 0.701(0.521, 0.934) | 1.646E-02 | 1.999E-02 |
| A2G2S1 | 0.404 | 0.15 | 1.498(1.121, 2.022) | 6.990E-03 | 9.141E-03 |
| FA2G2S1 | 0.832 | 0.173 | 2.298(1.660, 3.276) | 1.441E-06 | 8.166E-06 |
| FA2FG2S1 | -0.504 | 0.153 | 0.604(0.444, 0.810) | 9.861E-04 | 1.397E-03 |
| A2G2S2 | 0.324 | 0.149 | 1.383(1.036, 1.861) | 2.954E-02 | 3.139E-02 |

Initial glycans compositions: F at the start of the abbreviation indicates a core-fucose α 1,6-linked to the inner GlcNAc; Mx, number (x) of mannose on core GlcNAcs; Ax, number of antenna (GlcNAc) on trimannosyl core; A2, biantennary with both GlcNAcs as *β* 1,2-linked; B, bisecting GlcNAc linked β1,4 to β 1,3 mannose; G (x), number (x) of *β* 1,4 linked galactose on antenna; F (x), number (x) of fucose linked α 1,3 to antenna GlcNAc; S (x), number (x) of sialic acids linked to galactoses.CI, confidence interval; FDR, false discovery rate; GP, glycan peak; OR, odds ratio; SE, standard error; *β*, regression coefficient. Adjusted for Age, Gender, WBC, NEUT, ALT, and AST. *P* < 0.05 was considered statistically significant, *q* < 0.05 was considered statistically significant after correction using FDR

Table S6 Univariate and multivariate logistic regression analyses of four IgG N-glycosylation features.

|  | Univariate analysis | |  | Multivariate analysis | |
| --- | --- | --- | --- | --- | --- |
|  | OR (95% CI) | *q* |  | OR (95% CI) | *q* |
| Fucosylation | 1.261 (1.080, 1.493) | 5.556E-03 |  | 1.297 (1.102, 1.551) | 3.184E-03 |
| Bisecting GlcNAc | 0.857 (0.743, 0.981) | 2.921E-02 |  | 0.838 (0.716, 0.971) | 2.208E-02 |
| Sialylation | 1.176 (1.083, 1.285) | 5.703E-04 |  | 1.184 (1.084, 1.303) | 8.988E-04 |
| Galactosylation |  |  |  |  |  |
| G0 | 0.926 (0.882, 0.968) | 1.701E-03 |  | 0.919 (0.872, 0.964) | 1.800E-03 |
| G1 | 1.290 (1.123, 1.508) | 1.385E-03 |  | 1.288 (1.116, 1.518) | 1.800E-03 |
| G2 | 1.297 (1.162, 1.464) | 5.823E-05 |  | 1.335 (1.183, 1.528) | 5.281E-05 |

FDR, false discovery rate; GlcNAc, N-acetylglucosamine; G0, agalactosylation; G1, monogalactosylation; G2, digalactosylation; OR, odds ratio; SE, standard error; β, regression coefficient. Adjusted for Age, Gender, WBC, NEUT, ALT, and AST. *q* < 0.05 was considered statistically significant after correction using FDR

**Table S7** Correlation analysis between initial glycans and circulating inflammatory cytokines

| Variables | IL-2 | IL-4 | IL-6 | | IL-10 | TNF-α | IFN-γ |
| --- | --- | --- | --- | --- | --- | --- | --- |
| FA1 | -0.003 | 0.05 | 0.098 | 0.013 | | 0.028 | 0.036 |
| A2 | 0.026 | 0.09 | 0.071 | -0.001 | | -0.026 | -0.083 |
| A2B | -0.129 | -0.096 | 0.056 | -0.143 | | -0.007 | 0.061 |
| FA2 | -0.027 | 0.003 | 0.086 | -0.036 | | -0.06 | 0.05 |
| M5 | -0.049 | -0.088 | -0.007 | -0.129 | | 0.154* | 0.015 |
| FA2B | -0.033 | 0.02 | 0.061 | 0.05 | | -0.035 | -0.002 |
| A2G1 | -0.023 | 0.003 | 0.019 | -0.124 | | 0.028 | 0.012 |
| FA2[6]G1 | 0.044 | 0.044 | 0.074 | 0.132 | | 0.022 | 0.05 |
| FA2[3]G1 | -0.024 | -0.051 | -0.038 | -0.118 | | 0.105 | 0.031 |
| FA2[6]BG1 | -0.009 | 0.008 | 0.003 | 0.067 | | 0.041 | -0.012 |
| FA2[3]BG1 | -0.05 | -0.042 | -0.048 | -0.105 | | 0.065 | -0.018 |
| A2G2 | -0.027 | -0.055 | -0.05 | -0.062 | | -0.028 | -0.083 |
| A2BG2 | -0.044 | -0.09 | 0.009 | -0.112 | | 0.047 | -0.017 |
| FA2G2 | 0.036 | -0.02 | -0.058 | 0.009 | | 0.109 | 0.013 |
| FA2BG2 | -0.023 | -0.061 | -0.128 | 0.055 | | 0.017 | 0.02 |
| FA2G1S1 | 0.022 | 0.081 | 0.005 | -0.044 | | 0.089 | 0.017 |
| A2G2S1 | 0.034 | -0.05 | -0.143 | -0.069 | | -0.059 | -0.052 |
| FA2G2S1 | 0.028 | 0.021 | -0.126 | -0.064 | | 0.086 | -0.013 |
| FA2BG2S1 | -0.017 | -0.04 | -0.08 | 0.078 | | 0.018 | -0.135 |
| FA2FG2S1 | -0.024 | -0.038 | -0.189* | -0.016 | | -0.112 | -0.076 |
| A2G2S2 | 0.014 | -0.083 | -0.186* | -0.091 | | -0.107 | -0.023 |
| A2BG2S2 | 0.023 | 0.024 | -0.149* | -0.001 | | -0.088 | -0.099 |
| FA2G2S2 | 0.047 | -0.01 | -0.082 | 0.021 | | 0.018 | -0.035 |
| FA2BG2S2 | 0.069 | 0.094 | -0.058 | 0.068 | | 0.033 | -0.125 |

Initial glycans compositions: F at the start of the abbreviation indicates a core-fucose α 1,6-linked to the inner GlcNAc; Mx, number (x) of mannose on core GlcNAcs; Ax, number of antenna (GlcNAc) on trimannosyl core; A2, biantennary with both GlcNAcs as *β* 1,2-linked; B, bisecting GlcNAc linked β1,4 to β 1,3 mannose; G (x), number (x) of *β* 1,4 linked galactose on antenna; F (x), number (x) of fucose linked α 1,3 to antenna GlcNAc; S (x), number (x) of sialic acids linked to galactoses.GP, glycan peak; IL-2, interleukin-2; IL-4, interleukin-4; IL-6, interleukin-6; IL-10, interleukin-10; TNF-α, tumor necrosis factor-α. IFN-γ, interferon-γ; * Statistically significant at significant level of 0.05.

Table S8 Comparisons of 24 initial glycans between septic survivor and septic non-survivor

| Glycan peaks | Septic survivor  (n=121) | Septic non-survivor  (n=59) | *P* | *q* |
| --- | --- | --- | --- | --- |
| FA1 | 0.25 (0.19, 0.40) | 0.31 (0.25, 0.46) | 0.023 | 0.039 |
| A2 | 0.67 (0.39, 1.05) | 0.80 (0.56, 1.24) | 0.019 | 0.035 |
| A2B | 0.25 (0.18, 0.35) | 0.23 (0.18, 0.34) | 0.845 | 0.845 |
| FA2 | 24.22 (21.97, 27.38) | 30.40 (27.95, 33.52) | 2.017E-10 | 4.862E-09 |
| M5 | 0.13 (0.09, 0.19) | 0.10 (0.08, 0.15) | 0.012 | 0.029 |
| FA2B | 5.01 (4.26, 5.89) | 5.44 (4.72, 6.35) | 0.018 | 0.035 |
| A2G1 | 0.51 (0.34, 0.71) | 0.48 (0.35, 0.60) | 0.534 | 0.583 |
| FA2[6]G1 | 17.05 (16.21, 18.06) | 17.20 (15.58, 18.43) | 0.761 | 0.794 |
| FA2[3]G1 | 9.69 (9.15, 10.60) | 9.55 (8.49, 10.79) | 0.369 | 0.422 |
| FA2[6]BG1 | 4.42 (3.92, 5.01) | 4.23 (3.58, 4.67) | 0.031 | 0.049 |
| FA2[3]BG1 | 0.84 (0.73, 0.99) | 0.83 (0.69, 0.97) | 0.302 | 0.363 |
| A2G2 | 0.71 (0.49, 0.99) | 0.54 (0.40, 0.73) | 2.225E-03 | 0.010 |
| A2BG2 | 0.32 (0.25, 0.41) | 0.28 (0.21, 0.34) | 2.500E-03 | 0.010 |
| FA2G2 | 11.77 (10.09, 13.35) | 9.30 (7.50, 11.47) | 2.317E-06 | 1.854E-05 |
| FA2BG2 | 1.43 (1.23, 1.66) | 1.35 (1.12, 1.46) | 9.123E-03 | 0.024 |
| FA2G1S1 | 3.31 (2.87, 3.66) | 3.00 (2.60, 3.46) | 0.044 | 0.062 |
| A2G2S1 | 1.03 (0.88, 1.22) | 0.95 (0.85, 1.06) | 0.015 | 0.033 |
| FA2G2S1 | 9.06 (7.88, 10.60) | 7.18 (5.92, 8.43) | 5.797E-07 | 6.957E-06 |
| FA2BG2S1 | 2.23 (1.97, 2.56) | 2.16 (1.90, 2.39) | 0.156 | 0.196 |
| FA2FG2S1 | 0.17 (0.12, 0.24) | 0.13 (0.11, 0.17) | 5.470E-03 | 0.016 |
| A2G2S2 | 0.90 (0.74, 1.24) | 0.78 (0.66, 0.95) | 3.253E-03 | 0.011 |
| A2BG2S2 | 0.25 (0.20, 0.31) | 0.21 (0.17, 0.25) | 1.036E-03 | 6.218E-03 |
| FA2G2S2 | 1.93 (1.70, 2.46) | 1.84 (1.52, 2.15) | 0.077 | 0.102 |
| FA2BG2S2 | 2.53 (2.22, 2.84) | 2.26 (2.04, 2.76) | 0.036 | 0.054 |

Initial glycans compositions: F at the start of the abbreviation indicates a core-fucose α 1,6-linked to the inner GlcNAc; Mx, number (x) of mannose on core GlcNAcs; Ax, number of antenna (GlcNAc) on trimannosyl core; A2, biantennary with both GlcNAcs as β 1,2-linked; B, bisecting GlcNAc linked β1,4 to β 1,3 mannose; G (x), number (x) of β 1,4 linked galactose on antenna; F (x), number (x) of fucose linked α 1,3 to antenna GlcNAc; S (x), number (x) of sialic acids linked to galactoses.FDR, false discovery rate; GP, glycan peak. P < 0.05 was considered statistically significant; q < 0.05 was considered statistically significant after correction using FDR

Table S9 Comparisons of the 54 derived glycans between septic survivor and septic non-survivor

| Derived glycosylation traits | Septic survivor  (n=121) | Septic non-survivor  (n=59) | *P* | *q* |
| --- | --- | --- | --- | --- |
| **Sialylated glycans** |  |  |  |  |
| FGS/(FG+FGS) | 27.01 (24.60, 29.23) | 25.04 (23.57, 27.13) | 1.211E-03 | 5.448E-03 |
| FBGS/(FBG+FBGS) | 42.50 (37.64, 45.64) | 41.82 (38.78, 45.55) | 0.938 | 0.938 |
| FGS/(F+FG+FGS) | 18.17 (16.34, 20.66) | 15.34 (13.30, 17.18) | 7.761E-08 | 1.048E-06 |
| FBGS/(FB+FBG+FBGS) | 29.37 (25.72, 32.47) | 28.02 (24.73, 29.80) | 0.063 | 0.112 |
| FG1S1/(FG1+FG1S1) | 10.82 (9.87, 11.83) | 10.20 (9.10, 11.82) | 0.110 | 0.175 |
| FG2S1/(FG2+FG2S1+FG2S2) | 39.78 (38.02, 41.78) | 38.93 (37.38, 40.84) | 0.095 | 0.160 |
| FG2S2/(FG2+FG2S1+FG2S2) | 9.16 (7.66, 10.51) | 9.81 (8.10, 12.44) | 0.025 | 0.053 |
| FBG2S1/(FBG2+FBG2S1+FBG2S2) | 35.64 (32.80, 38.74) | 36.20 (34.68, 38.46) | 0.200 | 0.251 |
| FBG2S2/(FBG2+FBG2S1+FBG2S2) | 40.88 (38.02, 43.58) | 40.14 (37.53, 43.47) | 0.665 | 0.704 |
| F^total^S1/F^total^S2 | 3.18 (2.81, 3.46) | 2.96 (2.59, 3.36) | 0.035 | 0.073 |
| FS1/FS2 | 5.94 (5.32, 6.84) | 5.70 (4.73, 6.69) | 0.146 | 0.219 |
| FBS1/FBS2 | 0.89 (0.79, 1.00) | 0.89 (0.80, 1.00) | 0.556 | 0.608 |
| **Bisecting *N-*acetylglucosamine glycans** |  |  |  |  |
| FBS^total^/FS^total^ | 0.34 (0.30, 0.38) | 0.37 (0.32, 0.45) | 0.011 | 0.029 |
| FBS1/FS1 | 0.18 (0.16, 0.22) | 0.21 (0.17, 0.26) | 9.504-03 | 0.027 |
| FBS1/(FS1+FBS1) | 0.16 (0.14, 0.18) | 0.18 (0.15, 0.20) | 9.504-03 | 0.027 |
| FBS2/FS2 | 1.24 (1.08, 1.47) | 1.30 (1.13, 1.46) | 0.375 | 0.431 |
| FBS2/(FS2+FBS2) | 0.55 (0.52, 0.59) | 0.57 (0.53, 0.59) | 0.375 | 0.431 |
| **Neutral glycans** |  |  |  |  |
| FA1^n^ | 0.32 (0.25, 0.51) | 0.39 (0.31, 0.55) | 0.064 | 0.112 |
| A2^n^ | 0.85 (0.50, 1.30) | 1.02 (0.68, 1.51) | 0.041 | 0.081 |
| FA2^n^ | 31.40 (28.60, 34.11) | 36.72 (34.07, 41.22) | 1.205E-09 | 6.506E-08 |
| M5^n^ | 0.17 (0.12, 0.24) | 0.12 (0.10, 0.19) | 1.901E-03 | 7.895E-03 |
| FA2B^n^ | 6.32 (5.51, 7.43) | 6.50 (5.92, 7.73) | 0.118 | 0.182 |
| A2G1^n^ | 0.65 (0.44, 0.86) | 0.60 (0.42, 0.74) | 0.205 | 0.251 |
| FA2[6]G1^n^ | 21.85 (20.61, 23.35) | 21.22 (18.93, 22.43) | 0.011 | 0.029 |
| FA2[3]G1^n^ | 12.50 (11.44, 13.65) | 11.73 (10.32, 12.99) | 0.015 | 0.036 |
| FA2[6]BG1^n^ | 5.59 (5.05, 6.45) | 5.31 (4.36, 5.69) | 9.975E-04 | 5.387E-03 |
| FA2[3]BG1^n^ | 1.08 (0.91, 1.26) | 1.00 (0.84, 1.23) | 0.054 | 0.104 |
| A2G2^n^ | 0.89 (0.64, 1.23) | 0.65 (0.48, 0.89) | 3.309E-04 | 1.985E-03 |
| A2BG2 ^n^ | 0.40 (0.33, 0.52) | 0.34 (0.25, 0.40) | 1.846E-04 | 1.406E-03 |
| FA2G2 ^n^ | 14.70 (12.53, 17.04) | 11.23 (9.49, 14.33) | 1.772E-07 | 1.914E-06 |
| FA2BG2^n^ | 1.81 (1.58, 2.11) | 1.63 (1.33, 1.80) | 1.123E-03 | 5.448E-03 |
| **Galactosylated glycans** |  |  |  |  |
| G0^n^ | 39.25 (35.44, 42.59) | 44.92 (41.56, 50.57) | 1.467E-08 | 3.966E-07 |
| G1^n^ | 42.03 (40.71, 43.89) | 39.51 (37.26, 42.04) | 3.515E-06 | 3.163E-05 |
| G2^n^ | 18.20 (15.81, 20.76) | 13.93 (12.08, 17.57) | 3.918E-08 | 7.053E-07 |
| **Core fucosylated glycans** |  |  |  |  |
| F^n total^ | 96.54 (95.79, 97.31) | 96.88 (96.01, 97.36) | 0.235 | 0.282 |
| FG0^n total^/G0^n^ | 97.78 (96.87, 98.65) | 97.67 (96.77, 98.40) | 0.522 | 0.587 |
| FG1^n total^/G1^n^ | 98.47 (97.96, 98.97) | 98.45 (98.13, 98.82) | 0.790 | 0.805 |
| FG2^n total^ /G2^n^ | 92.39 (90.82, 94.00) | 92.61 (91.31, 94.29) | 0.563 | 0.608 |
| F^n^ | 81.51 (79.96, 82.95) | 82.33 (80.86, 83.45) | 0.063 | 0.112 |
| FG0^n^/G0^n^ | 81.34 (78.97, 83.31) | 82.91 (80.98, 84.49) | 0.016 | 0.036 |
| FG1^n^/G1^n^ | 82.28 (80.87, 83.77) | 82.92 (81.19, 84.48) | 0.205 | 0.251 |
| FG2^n^/G2^n^ | 81.55 (79.72, 84.08) | 81.52 (77.48, 83.49) | 0.172 | 0.238 |
| **Core fucosylation and bisecting *N-*acetylglucosamine** |  |  |  |  |
| FB^n^ | 14.85 (13.64, 16.55) | 14.46 (13.25, 15.85) | 0.191 | 0.251 |
| FBG0^n^/G0^n^ | 16.21 (14.45, 18.49) | 14.82 (13.34, 16.66) | 2.579E-03 | 9.948E-03 |
| FBG1^n^/G1^n^ | 16.01 (14.63, 17.81) | 15.98 (13.81, 17.55) | 0.194 | 0.251 |
| FBG2^n^/G2^n^ | 10.32 (8.59, 11.59) | 11.54 (9.62, 13.56) | 6.623E-03 | 0.024 |
| FB^n^/F^n^ | 18.36 (16.50, 20.73) | 17.60 (15.95, 19.53) | 0.156 | 0.221 |
| FB^n^/F^n total^ | 15.51 (14.17, 17.17) | 14.97 (13.76, 16.34) | 0.156 | 0.221 |
| F^n^/(B^n^ + FB^n^) | 5.32 (4.71, 5.81) | 5.55 (5.02, 6.08) | 0.101 | 0.165 |
| B^n^/(F^n^ + FB^n^) | 4.17 (3.35, 5.37) | 3.53 (2.60, 4.18) | 2.083E-04 | 1.406E-03 |
| FBG2^n^/FG2^n^ | 0.13 (0.10, 0.14) | 0.14 (0.12, 0.17) | 9.547E-03 | 0.027 |
| FBG2^n^ /(FG2^n^ + FBG2^n^) | 11.17 (9.32, 12.54) | 12.53 (10.41, 14.69) | 9.547E-03 | 0.027 |
| FG2^n^/(BG2^n^ + FBG2^n^) | 6.39 (5.54, 7.32) | 5.76 (4.80, 7.05) | 0.023 | 0.053 |
| BG2^n^/(FG2^n^ + FBG2^n^) | 25.64 (20.00, 32.57) | 26.07 (19.22, 33.24) | 0.716 | 0.743 |

B, bisecting *N*-acetylglucosamine (GlcNAc); F, fucose; FDR, false discovery rate; G, galactose; GP, glycan peak; S, Sialic acid. *P* < 0.05 was considered statistically significant; *q* < 0.05 was considered statistically significant after correction using FDR.

Table S10 Comparisons of the relative abundance of four IgG *N-*glycosylation features (%) between septic survivor and septic non-survivor

| Summary glycan traits | Septic survivor  (n=121) | Septic non-survivor  (n=59) | *P* | *q* |
| --- | --- | --- | --- | --- |
| Fucosylation | 95.34 (94.08, 96.22) | 95.68 (94.82, 97.27) | 0.017 | 0.026 |
| Bisecting GlcNAc | 17.18 (16.19, 18.62) | 16.85 (15.88, 18.02) | 0.262 | 0.262 |
| Sialylation | 21.98 (19.50, 24.66) | 18.76 (16.79, 21.20) | 2.915E-07 | 8.746E-07 |
| Galactosylation |  |  |  |  |
| G0 | 30.92 (27.70, 34.52) | 37.39 (34.73, 41.85) | 1.556E-09 | 9.333E-09 |
| G1 | 32.85 (31.99, 33.96) | 32.26 (30.51, 34.06) | 0.102 | 0.123 |
| G2 | 14.31 (12.56, 15.83) | 11.44 (9.80, 14.33) | 8.000E-07 | 1.600E-06 |

FDR, false discovery rate; GlcNAc, N-acetylglucosamine; G0, agalactosylation; G1, monogalactosylation; G2, digalactosylation.*P* < 0.05 was considered statistically significant; *q* < 0.05 was considered statistically significant after correction using FDR

Table S11 Characteristics of patients with Gram-negative sepsis and healthy controls

| Variables | Healthy controls  (n = 100) | Patients with Gram-negative sepsis  (n = 100) | *P* | *q* |
| --- | --- | --- | --- | --- |
| Age(years) | 65.00 (61.00, 72.00) | 69.50 (61.00, 77.00) | 0.109 | 0.119 |
| Gender [n(%)] |  |  | 0.119 | 0.119 |
| men | 40(40) | 52(52) |  |  |
| women | 60(60) | 48(48) |  |  |
| WBC (10*9/L) | 5.62 (4.73, 6.14) | 18.18 (11.80, 22.58) | 7.052E-30 | 2.821E-29 |
| NEUT (10*9/L) | 54.20 (51.70, 61.70) | 91.25 (87.27, 93.53) | 2.984E-33 | 2.387E-32 |
| PLT (10*9/L) | 241.00 (220.00, 256.50) | 186.00 (111.50, 270.75) | 1.407E-04 | 1.876E-04 |
| MPV (fL) | 9.50 (8.95, 10.00) | 10.00 (9.30, 11.53) | 1.007E-04 | 1.612E-04 |
| AST(U/L) | 21.70 (18.00, 24.70) | 40.15 (25.00, 109.60) | 2.503E-12 | 6.675E-12 |
| ALT(U/L) | 20.20 (15.20, 23.45) | 30.65 (19.67, 61.65) | 3.797E-07 | 7.595E-07 |
| SOFA | NA | 8.00 (6.00, 10.00) |  |  |
| 90-day outcome[n(%)] |  |  |  |  |
| Non-survivors | NA | 39(39) |  |  |
| Survivors | NA | 61(61) |  |  |

WBC, white blood cell; NEUT, absolute neutrophil count; PLT, platelet; MPV, Mean Platelet Volume: ALT, alanine aminotransferase; AST, aspartate aminotransferase; SOFA, Sequential Organ Failure Assessment; FDR, false discovery rate; *P* < 0.05 was considered statistically significant; *q* < 0.05 was considered statistically significant after correction using FDR

Table S12 Characteristics of patients with Gram-positive sepsis and healthy controls

| Variables | Healthy controls  (n = 100) | Patients with Gram-positive sepsis  (n = 80) | *P* | *q* |
| --- | --- | --- | --- | --- |
| Age(years) | 65.00 (61.00, 72.00) | 68.00 (60.00, 77.25) | 0.263 | 0.301 |
| Gender [n(%)] |  |  | 0.043 | 0.058 |
| men | 40(40) | 45(56.25) |  |  |
| women | 60(60) | 35(43.75) |  |  |
| WBC (10*9/L) | 5.62 (4.73, 6.14) | 11.43 (7.20, 17.60) | 7.372E-12 | 2.949E-11 |
| NEUT (10*9/L) | 54.20 (51.70, 61.70) | 88.15 (82.15, 93.35) | 1.236E-28 | 9.891E-28 |
| PLT (10*9/L) | 241.00 (220.00, 256.50) | 193.00 (106.25, 340.25) | 0.038 | 0.058 |
| MPV (fL) | 9.50 (8.95, 10.00) | 10.15 (9.38, 11.40) | 3.236E-04 | 8.631E-04 |
| AST(U/L) | 21.70 (18.00, 24.70) | 27.70 (17.67, 48.45) | 0.022 | 0.044 |
| ALT(U/L) | 20.20 (15.20, 23.45) | 20.90 (13.30, 34.90) | 0.488 | 0.488 |
| SOFA | NA | 8.00 (6.00, 10.25) |  |  |
| 90-day outcome[n(%)] |  |  |  |  |
| Non-survivors | NA | 20(25) |  |  |
| Survivors | NA | 60(75) |  |  |

WBC, white blood cell; NEUT, absolute neutrophil count; PLT, platelet; MPV, Mean Platelet Volume: ALT, alanine aminotransferase; AST, aspartate aminotransferase; SOFA, Sequential Organ Failure Assessment; FDR, false discovery rate; *P* < 0.05 was considered statistically significant; *q* < 0.05 was considered statistically significant after correction using FDR

Table S13 Levels of circulating inflammatory cytokines between patients with Gram-negative sepsis and healthy controls

| Inflammatory cytokines | Healthy controls  (n = 100) | Patients with Gram-  negative sepsis  (n = 100) | *P* | *q* |
| --- | --- | --- | --- | --- |
| IL-2 | 1.10 (0.01, 3.72) | 1.02 (0.53, 1.58) | 0.898 | 0.898 |
| IL-4 | 3.92 (1.58, 6.05) | 0.45 (0.00, 1.05) | 1.648E-08 | 2.471E-08 |
| IL-6 | 4.51 (2.33, 7.82) | 589.15(217.28, 3418.69) | 7.064E-18 | 4.239E-17 |
| IL-10 | 1.32 (0.53, 1.97) | 24.24 (5.71, 39.25) | 6.668E-16 | 2.000E-15 |
| TNF-α | 4.55 (3.47, 5.79) | 0.00 (0.00, 0.58) | 1.500E-13 | 2.999E-13 |
| IFN-γ | 1.89 (1.30, 3.93) | 0.84 (0.00, 3.52) | 0.012 | 0.015 |

FDR, false discovery rate; IL-2, interleukin-2; IL-4, interleukin-4; IL-6, interleukin-6; IL-10, interleukin-10; TNF-α, tumor necrosis factor-α. IFN-γ, interferon-γ; *P* < 0.05 was considered statistically significant; *q* < 0.05 was considered statistically significant after correction using FDR

Table S14 Levels of circulating inflammatory cytokines between patients with Gram-positive sepsis and healthy controls

| Inflammatory cytokines | Healthy controls  (n = 100) | Patients with Gram-  positive sepsis  (n = 100) | *P* | *q* |
| --- | --- | --- | --- | --- |
| IL-2 | 1.10 (0.01, 3.72) | 0.64 (0.31, 1.35) | 0.289 | 0.289 |
| IL-4 | 3.92 (1.58, 6.05) | 0.00 (0.00, 0.96) | 2.170E-10 | 3.255E-10 |
| IL-6 | 4.51 (2.33, 7.82) | 359.74 (132.75, 1960.59) | 7.064E-18 | 4.239E-17 |
| IL-10 | 1.32 (0.53, 1.97) | 16.06 (5.78, 37.62) | 3.978E-15 | 1.193E-14 |
| TNF-α | 4.55 (3.47, 5.79) | 0.00 (0.00, 0.90) | 1.528E-14 | 3.056E-14 |
| IFN-γ | 1.89 (1.30, 3.93) | 0.57 (0.00, 3.42) | 0.010 | 0.012 |

FDR, false discovery rate; IL-2, interleukin-2; IL-4, interleukin-4; IL-6, interleukin-6; IL-10, interleukin-10; TNF-α, tumor necrosis factor-α. IFN-γ, interferon-γ; *P* < 0.05 was considered statistically significant; *q* < 0.05 was considered statistically significant after correction using FDR

Table S15 Comparisons of 24 initial glycans between patients with Gram-negative sepsis and healthy controls

| Glycan peaks | Healthy controls  (n = 100) | Patients with Gram-negative sepsis  (n = 100) | *P* | *q* |
| --- | --- | --- | --- | --- |
| FA1 | 0.20 (0.11, 0.34) | 0.36 (0.25, 0.53) | 1.054E-08 | 2.529E-08 |
| A2 | 0.38 (0.25, 0.58) | 0.83 (0.52, 1.29) | 4.131E-12 | 1.982E-11 |
| A2B | 0.34 (0.23, 0.45) | 0.19 (0.14, 0.31) | 2.477E-06 | 4.247E-06 |
| FA2 | 22.50 (18.22, 25.04) | 27.42 (22.98, 32.59) | 1.947E-09 | 6.673E-09 |
| M5 | 0.35 (0.16, 0.48) | 0.10 (0.08, 0.14) | 1.090E-12 | 6.539E-12 |
| FA2B | 4.66 (3.86, 5.77) | 5.66 (4.61, 6.52) | 3.566E-06 | 5.706E-06 |
| A2G1 | 0.39 (0.24, 0.59) | 0.45 (0.32, 0.57) | 0.109 | 0.113 |
| FA2[6]G1 | 18.70 (17.41, 19.88) | 17.40 (16.28, 18.17) | 8.882E-07 | 1.640E-06 |
| FA2[3]G1 | 9.73 (8.51, 10.42) | 9.25 (7.99, 10.02) | 0.050 | 0.054 |
| FA2[6]BG1 | 4.04 (3.49, 4.58) | 4.29 (3.77, 4.79) | 0.050 | 0.054 |
| FA2[3]BG1 | 1.03 (0.75, 1.40) | 0.78 (0.69, 0.90) | 6.411E-06 | 9.617E-06 |
| A2G2 | 0.60 (0.39, 0.90) | 0.54 (0.38, 0.81) | 0.272 | 0.272 |
| A2BG2 | 0.44 (0.32, 0.62) | 0.28 (0.24, 0.35) | 4.295E-09 | 1.233E-08 |
| FA2G2 | 14.32 (12.32, 17.20) | 9.96 (7.75, 11.93) | 1.967E-17 | 4.150E-16 |
| FA2BG2 | 1.23 (1.04, 1.52) | 1.42 (1.32, 1.62) | 1.711E-04 | 2.415E-04 |
| FA2G1S1 | 3.01 (2.70, 3.41) | 3.27 (2.68, 3.60) | 0.050 | 0.054 |
| A2G2S1 | 0.74 (0.64, 0.88) | 0.96 (0.85, 1.07) | 4.583E-13 | 3.667E-12 |
| FA2G2S1 | 9.96 (8.09, 12.51) | 7.57 (6.17, 9.17) | 1.778E-09 | 6.673E-09 |
| FA2BG2S1 | 1.85 (1.65, 2.13) | 2.21 (1.98, 2.48) | 3.658E-08 | 7.981E-08 |
| FA2FG2S1 | 0.11 (0.07, 0.16) | 0.16 (0.13, 0.21) | 4.624E-09 | 1.233E-08 |
| A2G2S2 | 0.60 (0.41, 0.72) | 0.83 (0.73, 0.96) | 3.458E-17 | 4.150E-16 |
| A2BG2S2 | 0.18 (0.13, 0.24) | 0.22 (0.18, 0.28) | 2.282E-04 | 3.043E-04 |
| FA2G2S2 | 1.75 (1.46, 2.02) | 1.92 (1.69, 2.40) | 4.251E-03 | 5.370E-03 |
| FA2BG2S2 | 2.16 (1.75, 2.41) | 2.52 (2.14, 3.07) | 1.206E-07 | 2.412E-07 |

Initial glycans compositions: F at the start of the abbreviation indicates a core-fucose α 1,6-linked to the inner GlcNAc; Mx, number (x) of mannose on core GlcNAcs; Ax, number of antenna (GlcNAc) on trimannosyl core; A2, biantennary with both GlcNAcs as *β* 1,2-linked; B, bisecting GlcNAc linked β1,4 to β 1,3 mannose; G (x), number (x) of *β* 1,4 linked galactose on antenna; F (x), number (x) of fucose linked α 1,3 to antenna GlcNAc; S (x), number (x) of sialic acids linked to galactoses.FDR, false discovery rate; GP, glycan peak. *P* < 0.05 was considered statistically significant; *q* < 0.05 was considered statistically significant after correction using FDR

Table S16 Comparisons of the 54 derived glycans between patients with Gram-negative sepsis and healthy controls

| Derived glycosylation traits | Healthy controls  (n=100) | Patients with Gram-negative sepsis  (n = 100) | *P* | *q* |
| --- | --- | --- | --- | --- |
| **Sialylated glycans** |  |  |  |  |
| FGS/(FG+FGS) | 25.23 (23.09, 28.40) | 26.06 (23.98, 28.51) | 0.327 | 0.367 |
| FBGS/(FBG+FBGS) | 38.26 (34.98, 42.94) | 42.98 (39.52, 46.09) | 5.775E-05 | 1.155E-04 |
| FGS/(F+FG+FGS) | 18.34 (15.72, 22.24) | 16.67 (14.30, 18.75) | 9.426E-04 | 1.697E-03 |
| FBGS/(FB+FBG+FBGS) | 26.20 (23.21, 30.25) | 28.35 (25.24, 31.52) | 0.024 | 0.032 |
| FG1S1/(FG1+FG1S1) | 9.66 (8.62, 10.93) | 10.79 (9.65, 11.92) | 5.342E-05 | 1.109E-04 |
| FG2S1/(FG2+FG2S1+FG2S2) | 38.04 (35.94, 39.97) | 38.97 (37.55, 41.19) | 4.693E-04 | 8.738E-04 |
| FG2S2/(FG2+FG2S1+FG2S2) | 6.65 (5.58, 8.04) | 10.02 (9.02, 12.21) | 2.529E-20 | 2.732E-19 |
| FBG2S1/(FBG2+FBG2S1+FBG2S2) | 35.39 (31.97, 39.22) | 35.73 (32.95, 38.04) | 0.827 | 0.827 |
| FBG2S2/(FBG2+FBG2S1+FBG2S2) | 40.63 (35.80, 43.71) | 40.99 (37.96, 43.62) | 0.358 | 0.394 |
| F^total^S1/F^total^S2 | 3.87 (3.30, 4.44) | 2.84 (2.58, 3.24) | 4.496E-18 | 3.468E-17 |
| FS1/FS2 | 7.39 (6.22, 8.62) | 5.44 (4.82, 6.01) | 4.021E-15 | 2.413E-14 |
| FBS1/FBS2 | 0.89 (0.75, 1.06) | 0.88 (0.79, 0.98) | 0.500 | 0.519 |
| **Bisecting *N-*acetylglucosamine glycans** |  |  |  |  |
| FBS^total^/FS^total^ | 0.26 (0.23, 0.34) | 0.37 (0.33, 0.44) | 2.320E-13 | 1.253E-12 |
| FBS1/FS1 | 0.14 (0.12, 0.18) | 0.20 (0.18, 0.25) | 1.816E-12 | 7.003E-12 |
| FBS1/(FS1+FBS1) | 0.12 (0.10, 0.15) | 0.17 (0.15, 0.20) | 1.816E-12 | 7.003E-12 |
| FBS2/FS2 | 1.21 (1.03, 1.43) | 1.28 (1.09, 1.51) | 0.065 | 0.080 |
| FBS2/(FS2+FBS2) | 0.55 (0.51, 0.59) | 0.56 (0.52, 0.60) | 0.065 | 0.080 |
| **Neutral glycans** |  |  |  |  |
| FA1^n^ | 0.26 (0.14, 0.42) | 0.44 (0.30, 0.65) | 6.098E-09 | 1.829E-08 |
| A2^n^ | 0.49 (0.33, 0.74) | 1.06 (0.66, 1.59) | 2.532E-12 | 8.545E-12 |
| FA2^n^ | 28.21 (23.07, 31.07) | 34.27 (29.38, 40.05) | 1.179E-10 | 3.745E-10 |
| M5^n^ | 0.43 (0.21, 0.61) | 0.13 (0.10, 0.17) | 6.155E-13 | 3.022E-12 |
| FA2B^n^ | 5.93 (4.95, 6.97) | 7.11 (5.94, 7.94) | 1.084E-06 | 2.660E-06 |
| A2G1^n^ | 0.49 (0.31, 0.76) | 0.55 (0.40, 0.74) | 0.113 | 0.131 |
| FA2[6]G1^n^ | 23.71 (21.65, 25.06) | 21.77 (20.28, 23.40) | 2.482E-06 | 5.584E-06 |
| FA2[3]G1^n^ | 12.29 (10.83, 13.22) | 11.79 (9.98, 13.10) | 0.092 | 0.110 |
| FA2[6]BG1^n^ | 5.13 (4.41, 5.82) | 5.48 (4.67, 6.07) | 0.057 | 0.073 |
| FA2[3]BG1^n^ | 1.32 (0.92, 1.73) | 0.98 (0.87, 1.14) | 1.927E-06 | 4.524E-06 |
| A2G2^n^ | 0.77 (0.49, 1.15) | 0.68 (0.47, 1.02) | 0.214 | 0.277 |
| A2BG2 ^n^ | 0.55 (0.39, 0.79) | 0.36 (0.30, 0.45) | 8.769E-09 | 2.492E-08 |
| FA2G2 ^n^ | 18.18 (15.00, 22.23) | 12.29 (9.53, 15.36) | 3.577E-15 | 2.413E-14 |
| FA2BG2^n^ | 1.53 (1.29, 1.94) | 1.77 (1.61, 2.03) | 4.401E-04 | 8.487E-04 |
| **Galactosylated glycans** |  |  |  |  |
| G0^n^ | 34.99 (29.29, 39.33) | 43.12 (37.21, 49.26) | 2.403E-12 | 8.545E-12 |
| G1^n^ | 43.87 (41.05, 45.12) | 40.82 (37.71, 42.96) | 1.314E-05 | 2.838E-05 |
| G2^n^ | 21.26 (17.49, 26.75) | 15.35 (12.14, 18.29) | 9.278E-13 | 4.175E-12 |
| **Core fucosylated glycans** |  |  |  |  |
| F^n total^ | 96.43 (95.71, 97.45) | 96.71 (95.72, 97.56) | 0.537 | 0.547 |
| FG0^n total^/G0^n^ | 98.54 (97.97, 98.93) | 97.48 (96.34, 98.45) | 3.018E-08 | 7.761E-08 |
| FG1^n total^/G1^n^ | 98.87 (98.28, 99.29) | 98.62 (98.18, 99.02) | 8.592E-03 | 0.013 |
| FG2^n total^ /G2^n^ | 93.48 (91.95, 94.96) | 92.58 (90.94, 94.35) | 7.545E-03 | 0.017 |
| F^n^ | 82.24 (80.00, 84.17) | 81.92 (79.56, 83.30) | 0.050 | 0.066 |
| FG0^n^/G0^n^ | 80.48 (78.11, 83.05) | 81.59 (77.87, 83.31) | 0.394 | 0.426 |
| FG1^n^/G1^n^ | 83.91 (81.81, 85.65) | 82.84 (80.93, 84.51) | 8.654E-03 | 0.013 |
| FG2^n^/G2^n^ | 85.75 (83.44, 87.53) | 80.59 (77.48, 82.57) | 8.411E-20 | 7.570E-19 |
| **Core fucosylation and bisecting *N-*acetylglucosamine** |  |  |  |  |
| FB^n^ | 13.91 (12.52, 15.95) | 14.98 (13.56, 16.98) | 2.247E-03 | 3.914E-03 |
| FBG0^n^/G0^n^ | 17.89 (15.73, 19.98) | 16.16 (14.07, 18.88) | 4.610E-03 | 7.321E-03 |
| FBG1^n^/G1^n^ | 14.94 (13.22, 16.79) | 15.59 (14.19, 17.85) | 0.018 | 0.025 |
| FBG2^n^/G2^n^ | 7.75 (6.63, 8.78) | 12.12 (10.57, 13.83) | 3.743E-26 | 6.737E-25 |
| FB^n^/F^n^ | 16.90 (15.13, 19.83) | 18.58 (16.23, 21.27) | 3.276E-03 | 5.360E-03 |
| FB^n^/F^n total^ | 14.46 (13.14, 16.55) | 15.67 (13.97, 17.54) | 3.276E-03 | 5.360E-03 |
| F^n^/(B^n^ + FB^n^) | 5.65 (4.83, 6.32) | 5.23 (4.58, 6.00) | 0.020 | 0.028 |
| B^n^/(F^n^ + FB^n^) | 5.73 (4.03, 8.23) | 3.71 (3.06, 4.70) | 1.579E-08 | 4.262E-08 |
| FBG2^n^/FG2^n^ | 0.09 (0.08, 0.11) | 0.15 (0.13, 0.18) | 2.106E-26 | 5.686E-25 |
| FBG2^n^ /(FG2^n^ + FBG2^n^) | 8.36 (7.06, 9.51) | 13.16 (11.29, 15.29) | 2.106E-26 | 5.686E-25 |
| FG2^n^/(BG2^n^ + FBG2^n^) | 8.10 (6.83, 9.27) | 5.50 (4.68, 6.41) | 4.679E-22 | 6.317E-21 |
| BG2^n^/(FG2^n^ + FBG2^n^) | 28.24 (19.83, 38.54) | 26.03 (20.42, 33.64) | 0.404 | 0.428 |

B, bisecting *N*-acetylglucosamine (GlcNAc); F, fucose; FDR, false discovery rate; G, galactose; GP, glycan peak; S, Sialic acid. *P* < 0.05 was considered statistically significant; *q* < 0.05 was considered statistically significant after correction using FDR.

Table S17 Comparisons of the relative abundance of four IgG *N-*glycosylation features (%) between patients with Gram-negative sepsis and healthy controls

| Summary glycan traits | Healthy controls  (n = 100) | Patients with Gram-negative sepsis  (n = 100) | *P* | *q* |
| --- | --- | --- | --- | --- |
| Fucosylation | 95.83 (94.91, 96.42) | 95.09 (94.20, 96.02) | 1.891E-03 | 2.270E-03 |
| Bisecting GlcNAc | 15.86 (14.38, 17.39) | 17.38 (15.90, 19.45) | 2.881E-06 | 4.322E-06 |
| Sialylation | 20.14 (17.90, 23.41) | 19.57 (17.89, 22.32) | 0.178 | 0.178 |
| Galactosylation |  |  |  |  |
| G0 | 28.24 (23.38, 31.99) | 35.04 (29.24, 40.62) | 3.791E-10 | 1.137E-09 |
| G1 | 34.58 (32.59, 35.82) | 32.40 (30.68, 33.64) | 4.406E-07 | 8.812E-07 |
| G2 | 17.21 (14.09, 20.28) | 12.44 (9.88, 14.48) | 5.650E-15 | 3.390E-14 |

FDR, false discovery rate; GlcNAc, N-acetylglucosamine; G0, agalactosylation; G1, monogalactosylation; G2, digalactosylation.*P* < 0.05 was considered statistically significant; *q* < 0.05 was considered statistically significant after correction using FDR

Table S18 Comparisons of 24 initial glycans between patients with Gram-positive sepsis and healthy controls

| Glycan peaks | Healthy controls  (n = 100) | Patients with Gram-positive sepsis  (n = 80) | *P* | *q* |
| --- | --- | --- | --- | --- |
| FA1 | 0.20 (0.11, 0.34) | 0.25 (0.18, 0.31) | 0.080 | 0.101 |
| A2 | 0.38 (0.25, 0.58) | 0.61 (0.36, 0.90) | 4.080E-05 | 8.902E-05 |
| A2B | 0.34 (0.23, 0.45) | 0.28 (0.22, 0.36) | 0.054 | 0.077 |
| FA2 | 22.50 (18.22, 25.04) | 24.81 (22.87, 28.39) | 2.796E-06 | 7.457E-06 |
| M5 | 0.35 (0.16, 0.48) | 0.16 (0.11, 0.20) | 5.064E-09 | 4.051E-08 |
| FA2B | 4.66 (3.86, 5.77) | 4.91 (4.03, 5.44) | 0.829 | 0.829 |
| A2G1 | 0.39 (0.24, 0.59) | 0.57 (0.43, 0.77) | 9.905E-07 | 3.396E-06 |
| FA2[6]G1 | 18.70 (17.41, 19.88) | 16.95 (16.17, 18.06) | 3.399E-07 | 1.632E-06 |
| FA2[3]G1 | 9.73 (8.51, 10.42) | 10.21 (9.59, 11.08) | 1.142E-03 | 2.108E-03 |
| FA2[6]BG1 | 4.04 (3.49, 4.58) | 4.48 (3.89, 5.02) | 2.108E-03 | 3.613E-03 |
| FA2[3]BG1 | 1.03 (0.75, 1.40) | 0.90 (0.80, 1.04) | 0.101 | 0.121 |
| A2G2 | 0.60 (0.39, 0.90) | 0.73 (0.56, 0.99) | 7.291E-03 | 0.012 |
| A2BG2 | 0.44 (0.32, 0.62) | 0.34 (0.25, 0.42) | 7.654E-05 | 1.531E-04 |
| FA2G2 | 14.32 (12.32, 17.20) | 12.18 (10.90, 13.24) | 1.444E-07 | 8.662E-07 |
| FA2BG2 | 1.23 (1.04, 1.52) | 1.31 (1.09, 1.54) | 0.453 | 0.473 |
| FA2G1S1 | 3.01 (2.70, 3.41) | 3.16 (2.76, 3.55) | 0.194 | 0.222 |
| A2G2S1 | 0.74 (0.64, 0.88) | 1.10 (0.89, 1.32) | 2.630E-14 | 3.156E-13 |
| FA2G2S1 | 9.96 (8.09, 12.51) | 9.33 (8.08, 10.34) | 0.054 | 0.077 |
| FA2BG2S1 | 1.85 (1.65, 2.13) | 2.18 (1.87, 2.54) | 1.378E-06 | 4.133E-06 |
| FA2FG2S1 | 0.11 (0.07, 0.16) | 0.12 (0.07, 0.20) | 0.391 | 0.427 |
| A2G2S2 | 0.60 (0.41, 0.72) | 0.94 (0.70, 2.29) | 2.615E-15 | 6.277E-14 |
| A2BG2S2 | 0.18 (0.13, 0.24) | 0.24 (0.19, 0.42) | 5.541E-07 | 2.216E-06 |
| FA2G2S2 | 1.75 (1.46, 2.02) | 1.89 (1.59, 2.21) | 0.064 | 0.085 |
| FA2BG2S2 | 2.16 (1.75, 2.41) | 2.48 (2.16, 2.71) | 3.283E-05 | 7.879E-05 |

Initial glycans compositions: F at the start of the abbreviation indicates a core-fucose α 1,6-linked to the inner GlcNAc; Mx, number (x) of mannose on core GlcNAcs; Ax, number of antenna (GlcNAc) on trimannosyl core; A2, biantennary with both GlcNAcs as *β* 1,2-linked; B, bisecting GlcNAc linked β1,4 to β 1,3 mannose; G (x), number (x) of *β* 1,4 linked galactose on antenna; F (x), number (x) of fucose linked α 1,3 to antenna GlcNAc; S (x), number (x) of sialic acids linked to galactoses. FDR, false discovery rate; GP, glycan peak. *P* < 0.05 was considered statistically significant; *q* < 0.05 was considered statistically significant after correction using FDR

Table S19 Comparisons of the 54 derived glycans between patients with Gram-positive sepsis and healthy controls

| Derived glycosylation traits | Healthy controls  (n=100) | Patients with Gram-positive sepsis  (n = 80) | *P* | *q* |
| --- | --- | --- | --- | --- |
| **Sialylated glycans** |  |  |  |  |
| FGS/(FG+FGS) | 25.23 (23.09, 28.40) | 26.92 (24.78, 28.77) | 0.081 | 0.110 |
| FBGS/(FBG+FBGS) | 38.26 (34.98, 42.94) | 41.09 (36.79, 44.33) | 0.046 | 0.069 |
| FGS/(F+FG+FGS) | 18.34 (15.72, 22.24) | 18.09 (16.47, 20.21) | 0.392 | 0.441 |
| FBGS/(FB+FBG+FBGS) | 26.20 (23.21, 30.25) | 28.76 (24.97, 32.49) | 0.011 | 0.018 |
| FG1S1/(FG1+FG1S1) | 9.66 (8.62, 10.93) | 10.47 (9.30, 11.63) | 0.040 | 0.062 |
| FG2S1/(FG2+FG2S1+FG2S2) | 38.04 (35.94, 39.97) | 40.15 (37.95, 41.74) | 1.757E-05 | 5.655E-05 |
| FG2S2/(FG2+FG2S1+FG2S2) | 6.65 (5.58, 8.04) | 8.06 (7.21, 9.32) | 2.215E-07 | 2.392E-06 |
| FBG2S1/(FBG2+FBG2S1+FBG2S2) | 35.39 (31.97, 39.22) | 36.94 (33.89, 40.13) | 0.060 | 0.086 |
| FBG2S2/(FBG2+FBG2S1+FBG2S2) | 40.63 (35.80, 43.71) | 40.31 (37.15, 43.41) | 0.991 | 0.991 |
| F^total^S1/F^total^S2 | 3.87 (3.30, 4.44) | 3.36 (3.14, 3.66) | 2.052E-05 | 6.157E-05 |
| FS1/FS2 | 7.39 (6.22, 8.62) | 6.43 (5.87, 7.26) | 4.374E-04 | 9.448E-04 |
| FBS1/FBS2 | 0.89 (0.75, 1.06) | 0.91 (0.82, 1.03) | 0.410 | 0.452 |
| **Bisecting *N-*acetylglucosamine glycans** |  |  |  |  |
| FBS^total^/FS^total^ | 0.26 (0.23, 0.34) | 0.33 (0.29, 0.37) | 3.910E-06 | 1.920E-05 |
| FBS1/FS1 | 0.14 (0.12, 0.18) | 0.18 (0.15, 0.22) | 8.507E-06 | 3.062E-05 |
| FBS1/(FS1+FBS1) | 0.12 (0.10, 0.15) | 0.15 (0.13, 0.18) | 8.507E-06 | 3.062E-05 |
| FBS2/FS2 | 1.21 (1.03, 1.43) | 1.26 (1.13, 1.41) | 0.248 | 0.291 |
| FBS2/(FS2+FBS2) | 0.55 (0.51, 0.59) | 0.56 (0.53, 0.59) | 0.248 | 0.291 |
| **Neutral glycans** |  |  |  |  |
| FA1^n^ | 0.26 (0.14, 0.42) | 0.31 (0.24, 0.39) | 0.075 | 0.103 |
| A2^n^ | 0.49 (0.33, 0.74) | 0.74 (0.47, 1.10) | 2.047E-05 | 8.661E-05 |
| FA2^n^ | 28.21 (23.07, 31.07) | 31.76 (29.47, 34.41) | 7.734E-08 | 1.392E-06 |
| M5^n^ | 0.43 (0.21, 0.61) | 0.20 (0.13, 0.26) | 3.763E-09 | 1.679E-07 |
| FA2B^n^ | 5.93 (4.95, 6.97) | 5.95 (5.32, 6.51) | 0.941 | 0.959 |
| A2G1^n^ | 0.49 (0.31, 0.76) | 0.72 (0.53, 0.95) | 7.756E-07 | 5.983E-06 |
| FA2[6]G1^n^ | 23.71 (21.65, 25.06) | 21.26 (19.91, 22.85) | 6.218E-09 | 1.679E-07 |
| FA2[3]G1^n^ | 12.29 (10.83, 13.22) | 12.81 (12.02, 13.83) | 2.741E-03 | 5.104E-03 |
| FA2[6]BG1^n^ | 5.13 (4.41, 5.82) | 5.55 (5.04, 6.31) | 3.155E-03 | 5.680E-03 |
| FA2[3]BG1^n^ | 1.32 (0.92, 1.73) | 1.20 (0.96, 1.34) | 0.087 | 0.115 |
| A2G2^n^ | 0.77 (0.49, 1.15) | 0.94 (0.72, 1.23) | 0.013 | 0.021 |
| A2BG2 ^n^ | 0.55 (0.39, 0.79) | 0.42 (0.30, 0.54) | 1.012E-04 | 2.483E-04 |
| FA2G2 ^n^ | 18.18 (15.00, 22.23) | 15.19 (13.58, 16.79) | 1.630E-06 | 9.781E-06 |
| FA2BG2^n^ | 1.53 (1.29, 1.94) | 1.67 (1.31, 1.92) | 0.494 | 0.513 |
| **Galactosylated glycans** |  |  |  |  |
| G0^n^ | 34.99 (29.29, 39.33) | 38.87 (36.35, 41.99) | 9.958E-07 | 6.722E-06 |
| G1^n^ | 43.87 (41.05, 45.12) | 41.83 (40.71, 43.66) | 1.257E-03 | 2.610E-03 |
| G2^n^ | 21.26 (17.49, 26.75) | 18.45 (16.18, 19.86) | 4.547E-05 | 1.169E-04 |
| **Core fucosylated glycans** |  |  |  |  |
| F^n total^ | 96.43 (95.71, 97.45) | 96.55 (95.84, 97.18) | 0.455 | 0.482 |
| FG0^n total^/G0^n^ | 98.54 (97.97, 98.93) | 97.97 (97.19, 98.74) | 4.742E-03 | 8.002E-03 |
| FG1^n total^/G1^n^ | 98.87 (98.28, 99.29) | 98.28 (97.72, 98.75) | 1.346E-07 | 1.817E-06 |
| FG2^n total^ /G2^n^ | 93.48 (91.95, 94.96) | 92.48 (91.08, 93.63) | 1.504E-03 | 2.901E-03 |
| F^n^ | 82.24 (80.00, 84.17) | 82.03 (80.53, 83.22) | 0.350 | 0.402 |
| FG0^n^/G0^n^ | 80.48 (78.11, 83.05) | 82.81 (80.08, 85.01) | 3.755E-04 | 8.449E-04 |
| FG1^n^/G1^n^ | 83.91 (81.81, 85.65) | 81.97 (80.94, 83.28) | 4.169E-05 | 1.126E-04 |
| FG2^n^/G2^n^ | 85.75 (83.44, 87.53) | 83.37 (81.35, 85.29) | 2.917E-06 | 1.575E-05 |
| **Core fucosylation and bisecting *N-*acetylglucosamine** |  |  |  |  |
| FB^n^ | 13.91 (12.52, 15.95) | 14.43 (13.48, 15.77) | 0.237 | 0.291 |
| FBG0^n^/G0^n^ | 17.89 (15.73, 19.98) | 15.20 (14.01, 16.76) | 3.726E-07 | 3.353E-06 |
| FBG1^n^/G1^n^ | 14.94 (13.22, 16.79) | 16.30 (14.86, 17.60) | 1.348E-03 | 2.696E-03 |
| FBG2^n^/G2^n^ | 7.75 (6.63, 8.78) | 8.99 (7.71, 10.08) | 1.780E-05 | 5.655E-05 |
| FB^n^/F^n^ | 16.90 (15.13, 19.83) | 17.54 (16.31, 19.46) | 0.217 | 0.278 |
| FB^n^/F^n total^ | 14.46 (13.14, 16.55) | 14.92 (14.03, 16.29) | 0.217 | 0.278 |
| F^n^/(B^n^ + FB^n^) | 5.65 (4.83, 6.32) | 5.53 (5.02, 5.86) | 0.451 | 0.482 |
| B^n^/(F^n^ + FB^n^) | 5.73 (4.03, 8.23) | 4.41 (3.11, 5.56) | 1.166E-04 | 2.737E-04 |
| FBG2^n^/FG2^n^ | 0.09 (0.08, 0.11) | 0.11 (0.09, 0.12) | 5.592E-06 | 2.323E-05 |
| FBG2^n^ /(FG2^n^ + FBG2^n^) | 8.36 (7.06, 9.51) | 9.97 (8.39, 10.92) | 5.592E-06 | 2.323E-05 |
| FG2^n^/(BG2^n^ + FBG2^n^) | 8.10 (6.83, 9.27) | 7.17 (6.39, 8.54) | 4.392E-03 | 7.651E-03 |
| BG2^n^/(FG2^n^ + FBG2^n^) | 28.24 (19.83, 38.54) | 25.11 (18.67, 31.23) | 5.687E-02 | 8.300E-02 |

B, bisecting *N*-acetylglucosamine (GlcNAc); F, fucose; FDR, false discovery rate; G, galactose; GP, glycan peak; S, Sialic acid. *P* < 0.05 was considered statistically significant; *q* < 0.05 was considered statistically significant after correction using FDR.

Table S20 Comparisons of the relative abundance of four IgG *N-*glycosylation features (%) between patients with Gram-positive sepsis and healthy controls

| Summary glycan traits | Healthy controls  (n = 100) | Patients with Gram-positive sepsis  (n = 80) | *P* | *q* |
| --- | --- | --- | --- | --- |
| Fucosylation | 95.83 (94.91, 96.42) | 95.95 (94.26, 97.71) | 0.191 | 0.191 |
| Bisecting GlcNAc | 15.86 (14.38, 17.39) | 16.87 (16.18, 17.72) | 8.751E-04 | 1.750E-03 |
| Sialylation | 22.41 (19.84, 25.11) | 22.22 (19.63, 24.69) | 0.014 | 0.017 |
| Galactosylation |  |  |  |  |
| G0 | 28.24 (23.38, 31.99) | 30.91 (28.44, 34.72) | 4.866E-05 | 1.460E-04 |
| G1 | 34.58 (32.59, 35.82) | 33.31 (32.28, 34.66) | 8.294E-03 | 0.012 |
| G2 | 17.21 (14.09, 20.28) | 14.55 (12.90, 15.77) | 4.161E-06 | 2.497E-05 |

FDR, false discovery rate; GlcNAc, N-acetylglucosamine; G0, agalactosylation; G1, monogalactosylation; G2, digalactosylation.*P* < 0.05 was considered statistically significant; *q* < 0.05 was considered statistically significant after correction using FDR


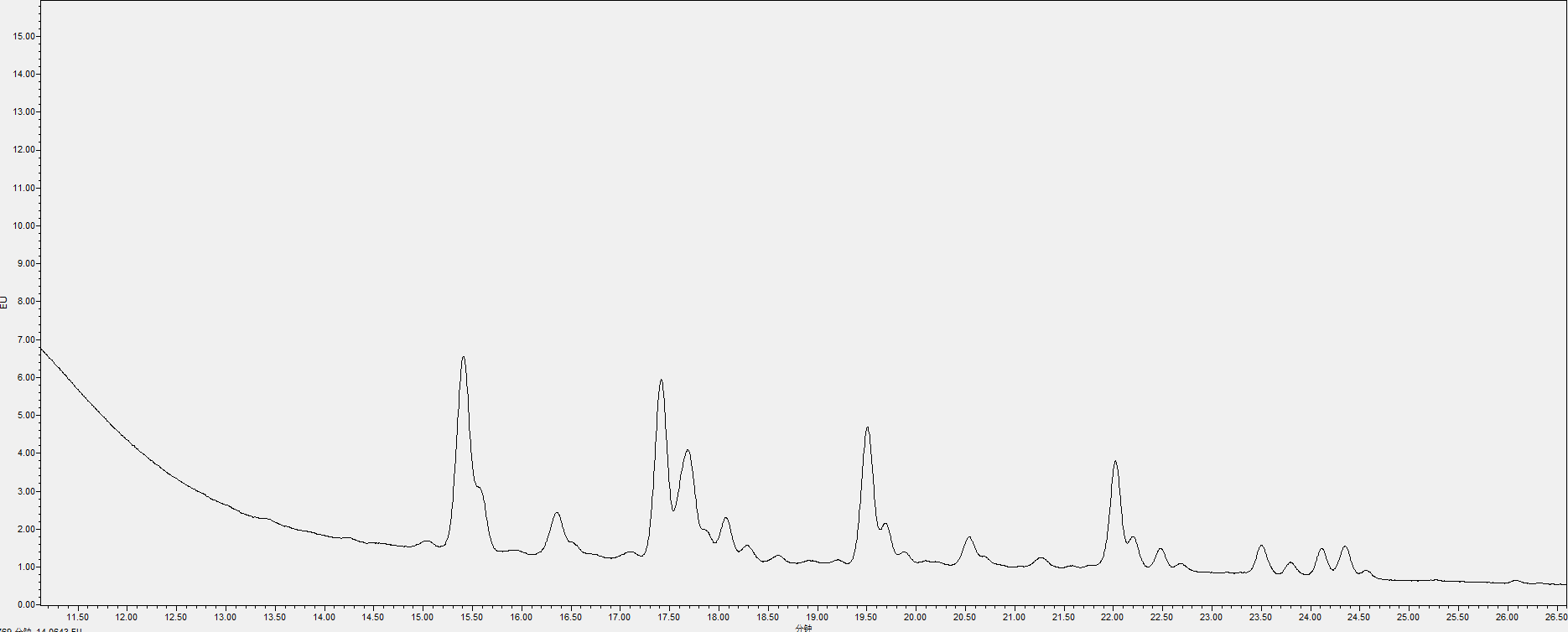


Fig. S1 A chromatogram showing the distribution of N-glycan peaks in a healthy control


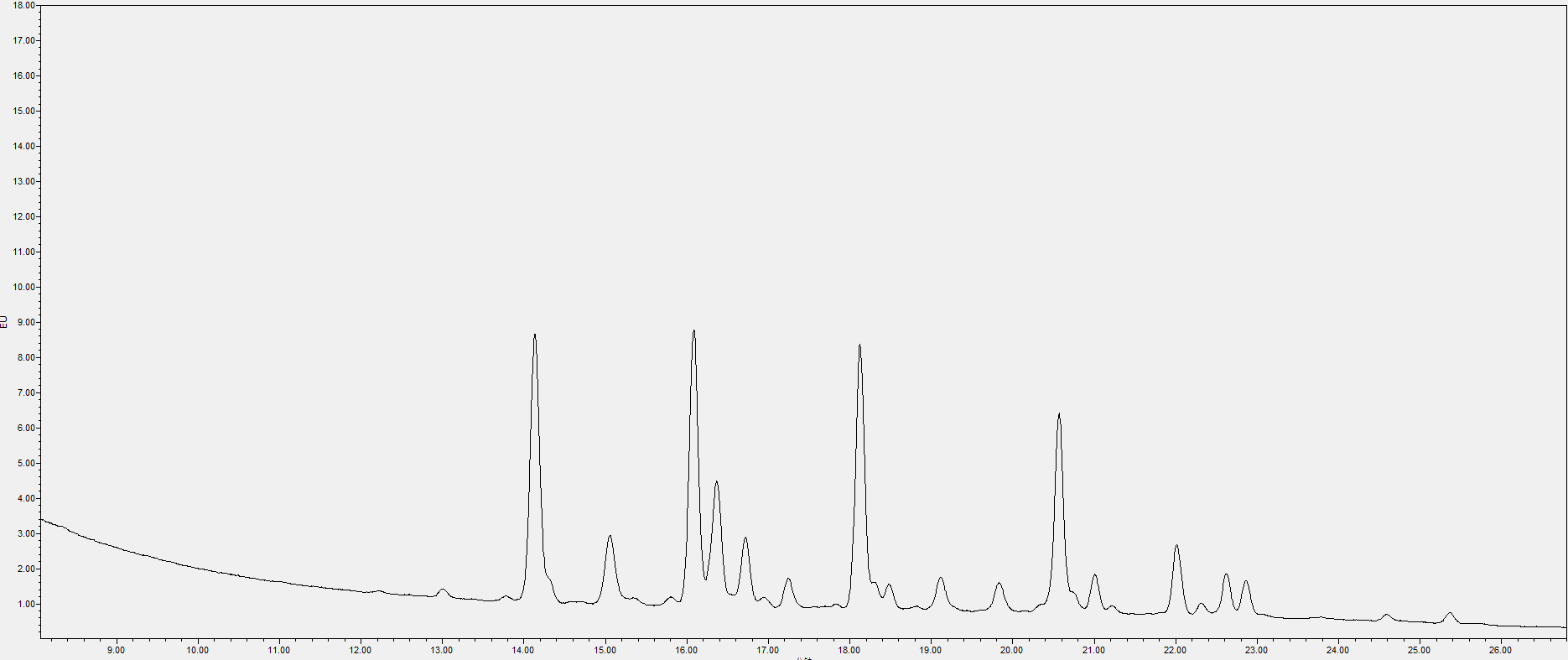


Fig. S2 A chromatogram showing the distribution of N-glycan peaks in a patient with Gram-positive sepsis


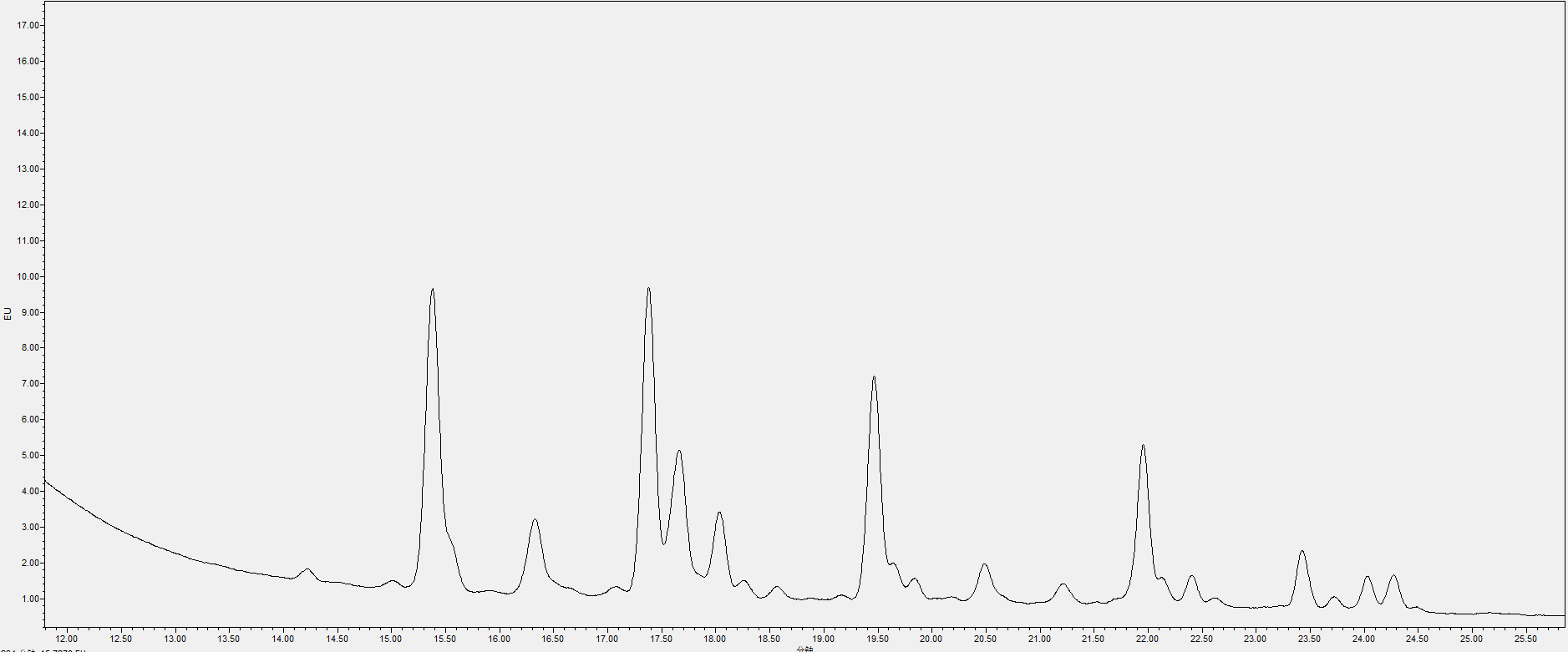


Fig. S3 A chromatogram showing the distribution of N-glycan peaks in a patient with Gram-negative sepsis


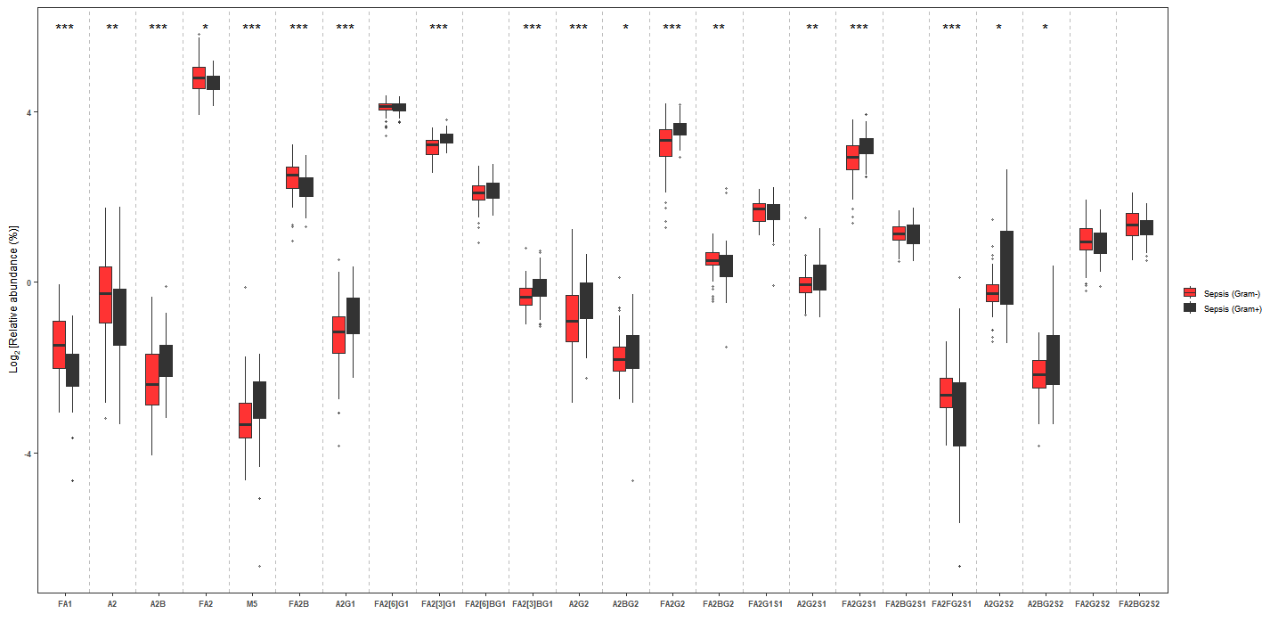


Fig. S4 Comparison of the differences in IgG *N*-initial glycan peaks in patients with sepsis. Initial glycans compositions: F at the start of the abbreviation indicates a core-fucose α 1,6-linked to the inner GlcNAc; Mx, number (x) of mannose on core GlcNAcs; Ax, number of antenna (GlcNAc) on trimannosyl core; A2, biantennary with both GlcNAcs as β 1,2-linked; B, bisecting GlcNAc linked β1,4 to β 1,3 mannose; G (x), number (x) of β 1,4 linked galactose on antenna; F (x), number (x) of fucose linked α 1,3 to antenna GlcNAc; S (x), number (x) of sialic acids linked to galactoses. *P < 0.05; ** P < 0.01; *** P < 0.001

Fig. S5 Comparisons of the relative abundance of four IgG *N*-glycosylation features (%) between in patients with sepsis. Fuc, Fucosylation; Bis, Bisecting GlcNAc; Sia, Sialylation; G0, agalactosylation; G1, monogalactosylation; G2, digalactosylation


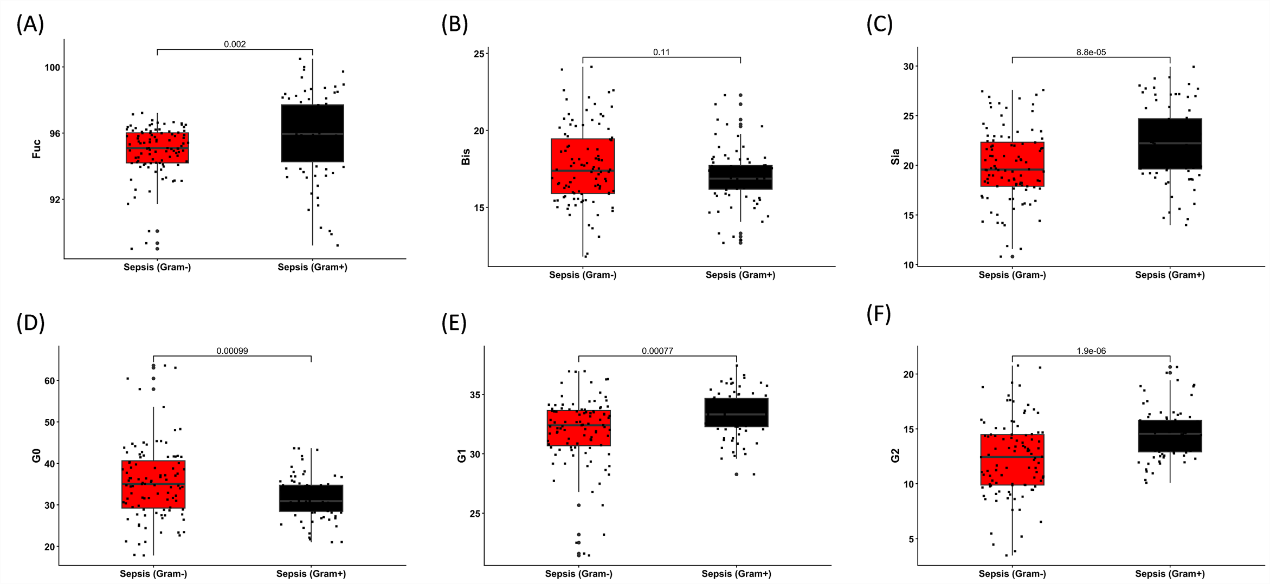


Fig. S6 LASSO regression analysis for variable selection. (A) LASSO coefficient of 24 variables; (B) optimal penalty coefficient (λ = 0.07864528) in LASSO regression. LASSO, least absolute shrinkage and selection operator


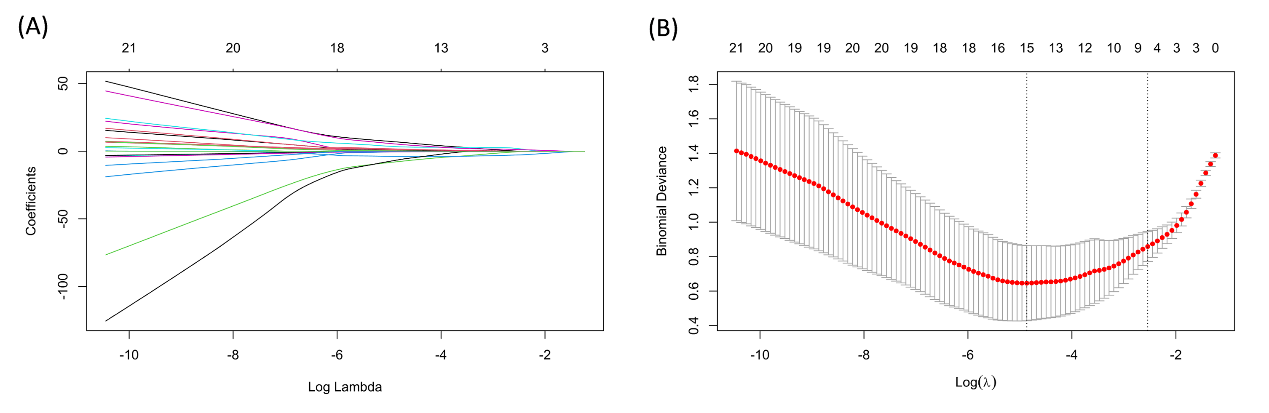


Fig. S7 LASSO regression analysis for variable selection. (A) LASSO coefficient of 24 variables; (B) optimal penalty coefficient (λ = 0.02195309) in LASSO regression. LASSO, least absolute shrinkage and selection operator


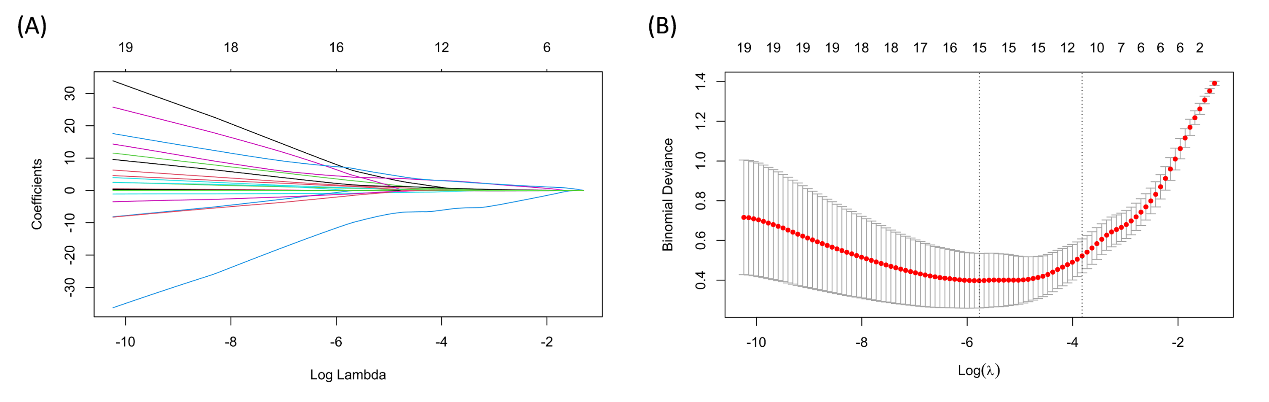


Fig. S8 Receiver operating characteristic (ROC) curve analysis for glycan-based prediction models. (A) Gram-negative group versus healthy control group; (B) Gram-positive group versus healthy control group.
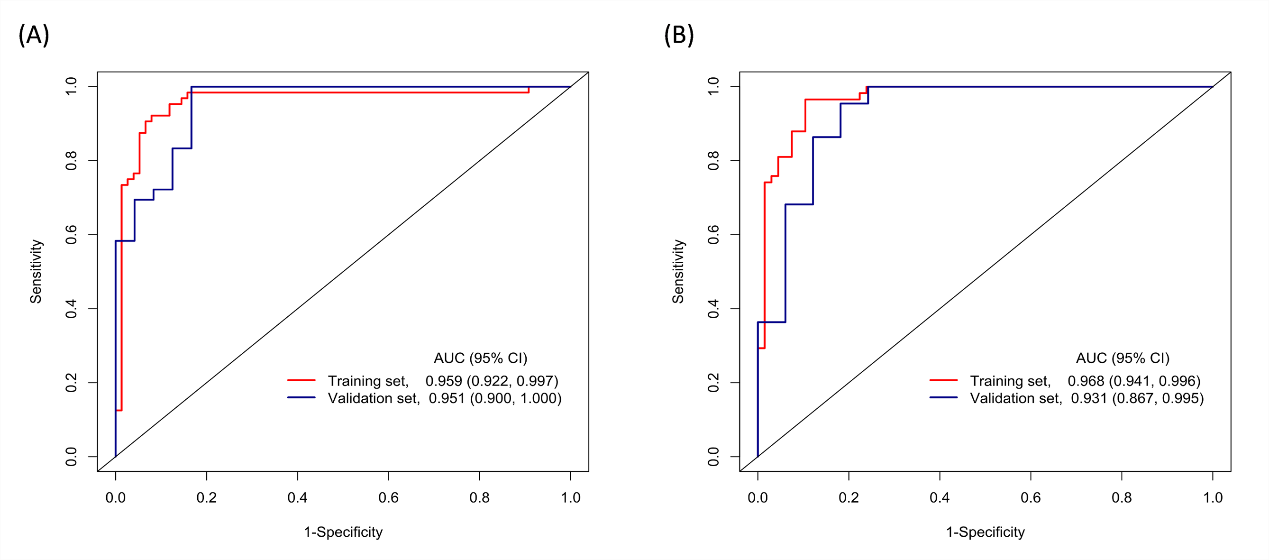

Supplement: Supplementary file 1 [file DataSheet1.docx]
